# Metabolomic and transcriptomic analyses identify external conditions and key genes underlying high levels of toxic glycoalkaloids in tubers of stress-sensitive potato cultivars

**DOI:** 10.3389/fpls.2023.1210850

**Published:** 2023-10-04

**Authors:** Irene Merino, Alexandra Olarte Guasca, Ales Krmela, Usman Arif, Ashfaq Ali, Erik Westerberg, Siddhi Kashinanth Jalmi, Jana Hajslova, Vera Schulzova, Folke Sitbon

**Affiliations:** ^1^ Department of Plant Biology, Uppsala BioCenter, Swedish University of Agricultural Sciences, and Linnean Centre for Plant Biology, Uppsala, Sweden; ^2^ Department of Food Analysis and Nutrition, University of Chemistry and Technology Prague, Prague, Czechia; ^3^ National Bioinformatics Infrastructure Sweden (NBIS), SciLifeLab at Department of Immunotechnology, Lund University, Lund, Sweden

**Keywords:** abiotic stress, calystegines, glycoalkaloids, metabolomics, potato (*Solanum tuberosum* L.), solanine, transcriptomics

## Abstract

**Introduction:**

High levels of toxic steroidal glycoalkaloids (SGAs) in potato tubers constitute a recognized food quality problem. Tuber SGA levels vary between potato cultivars and can increase after post-harvest stresses such as wounding and light exposure. A few cultivars, e.g., ‘Magnum Bonum’ and ‘Lenape,’ have been withdrawn from commercial sales due to excessive SGA levels during some cultivation years. However, these sudden SGA increases are diffucult to predict, and their causes are not understood. To identify external and genetic factors that underlie sudden SGA increases in certain potato cultivars, we have here in a 2-year study investigated ‘Magnum Bonum’ and five additional table potato cultivars for their SGA levels after wounding and light exposure.

**Results and methods:**

Results showed that ‘Magnum Bonum’ has an unusual strong SGA response to light exposure, but not to wounding, whereas ‘Bintje’ displayed an opposite regulation. Levels of calystegine alkaloids were not significantly altered by treatments, implicating independent metabolic regulation of SGA and calystegine levels also under conditions of high SGA accumulation. Metabolomic and transcriptomic analyses identified a small number of key genes whose expression correlated with SGA differences between cultivars. Overexpression of two key genes in transgenic low-SGA potato cultivars increased their leaf SGA levels significantly.

**Discussion:**

The results show that a strong response to light can underlie the SGA peaks that occasionally occur in certain potato cultivars and indicate that a between-cultivar variation in the expression of single SGA key genes can account for cultivar SGA differerences. We propose that current attempts to mitigate the SGA hazard will benefit from an increased consideration of cultivar-dependent SGA responses to post-harvest conditions, particularly light exposure. The identified key SGA genes can now be used as a molecular tool in this work.

## Introduction

Steroidal glycoalkaloids (SGAs) are bitter-tasting neurotoxic compounds that are naturally present in several plant species within the Solanaceae family, including major crop species such as the eggplant (*Solanum melongea*), tomato (*Solanum lycopersicum*), and potato (*Solanum tuberosum*) (Friedman and McDonald, 1997; Ghisalberti, 2006; Ginzberg et al., 2009; Zhao et al., 2021). In cultivated potatoes, the main SGAs are denoted α-solanine and α-chaconine. They are derived from the same aglucone, solanidine, but differ in their composition of a tri-saccharide moiety. The sum of α-solanine and α-chaconine usually accounts for over 95% of the total tuber SGA (Friedman and Levin, 2016). Depending on the cultivar, minor SGAs may be, e.g., solamarine, solamargine, solasonine, and demissine. Wild potato species have a broader SGA composition (Kozukue et al., 2008; Distl and Wink, 2009).

The SGA levels in potato plants are generally highest in flowers, berries, and sprouts, whereas the levels in tubers are lower. However, certain stresses and environmental factors, such as wounding or light exposure, can increase tuber SGA levels significantly (Sinden et al., 1984; Friedman and McDonald, 1999). These SGA increases vary between both stresses and cultivars (Petersson et al., 2013a), indicating a complex interaction between the environment and genotype.

SGAs are formed from cholesterol, one of the minor sterols in plant sterol metabolism (Ohyama et al., 2013; Petersson et al., 2013b). Cholesterol is converted into SGA in a series of SGA-specific reactions including hydroxylations, glycosylations, and a transamination step (Cárdenas et al., 2015). A separation between sterol syntheses for housekeeping, or SGA, purposes is mediated through duplications of a small set of differentially regulated sterol-biosynthetic genes, giving two parallel routes to cholesterol (Sonawane et al., 2016).

Many of the genes that encode SGA-biosynthetic enzymes are co-regulated (Cárdenas et al., 2016; Nahar et al., 2017; Shen et al., 2022). The APETALA2/Ethylene Response Factor GLYCOALKALOID METABOLISM9 (GAME9, also denoted JRE4) is central in this regulation, either alone or in co-operation with the transcription factor MYC2 (Cárdenas et al., 2016; Thagun et al., 2016; Swinnen et al., 2022). An outline of the potato sterol and SGA biosynthesis is given in **Figure S1**. The main genes/proteins involved and their corresponding abbreviations in the present study are listed in **Table 1** and **Table S1**, respectively.

**Table 1:** Abbreviations of sterol/SGA-biosynthetic enzymes, used in the present investigation.

Abbreviation	Also denoted	Enzymatic function
HMGS		3-Hydroxy-3-methylglutaryl-coenzyme A synthase
HMGR		3-Hydroxy-3-methylglutaryl-coenzyme A reductase
MVK		Mevalonate kinase
PMVK		Phosphomevalonate kinase
MVDPD		Mevalonate diphosphate decarboxylase
IDI	IPPI	Isopentenyl diphosphate isomerase (IPP isomerase)
FPS		Farnesyl pyrophosphate synthase
SQS		Squalene synthase
SQE		Squalene epoxidase (monooxygenase)
CAS/LAS		Cycloartenol/lanosterol synthase
DWF1	SSR1	Sterol side-chain Δ24-reductase
DWF1-L	SSR2	Sterol side-chain Δ24-reductase-like
SMO1		Sterol methyl-oxidase 1-2
SMO1-L		Sterol methyl-oxidase 1-like
SMO2		Sterol C4 methyl oxidase 2
SMO2-L		Sterol C4 methyl oxidase 2-like
SMT1		Sterol C24-methyltransferase 1
SMT2		Sterol C24-methyltransferase 2
DWF5		Δ7 Sterol reductase
DWF5-L		Δ7 Sterol reductase-like
DWF7	C5-SD1	Δ7-C5 sterol desaturase
DWF7-L	C5-SD2	Δ7-C5 sterol desaturase-like
CPI		Cyclopropyl isomerase
SI	HYDRA	Δ8-Δ7 Sterol isomerase
FK	FACKEL	Δ14- Sterol reductase
CYP51		Obtusifoliol C14-demethylase
C22-desat.	CYP710A	Stigmasterol synthase
3β-HSD		3β-Hydroxysteroid-dehydrogenase/decarboxylase
CYP72A188	GAME6; PGA2	Steroid C22-oxygenase
CYP72A208a	GAME8a	Cytochrome P450
CYP72A208b	GAME8b; PGA1	Steroid C26-oxygenase
CYP72A186	GAME7	Cytochrome P450
CYP88B1	GAME4	Cholesterol C26-oxidase
2-ODD (GAME11)	GAME11; 16DOX	2-Oxoglutarate-dependent dioxygenase (GAME11)
2-ODD (DPS)	DPS	2-Oxoglutarate-dependent dioxygenase (DPS)
2-ODD (GAME31)	GAME31	2-Oxoglutarate-dependent dioxygenase (GAME31)
TAM iso1		GABA transaminase isoform 1
TAM iso2	GAME12; PGA4	GABA transaminase isoform 2 (TAM2)
TAM iso3		GABA transaminase isoform 3
SGT1		UDP-galactose:solanidine galactosyltransferase
SGT2		UDP-glucose:solanidine glucosyltransferase
SGT3		β-Solanine/β-chaconine rhamnosyltransferase
GAME9	JRE4	Apetala/Ethylene response transcription factor

An “-L” denotes proteins with a special role in cholesterol synthesis and being regulated by wound/light. A full list with corresponding potato genes is given in **Table S1**.

The toxicity of different SGAs varies depending on their chemical structure, and α-solanine and α-chaconine are considered as being among the more toxic forms (Friedman and McDonald, 1997; Milner et al., 2011). For food safety reasons, an upper limit of 200 mg total SGA kg^−1^ fresh weight (FW), as measured in raw unpeeled tubers is widely recommended in potatoes aimed for human consumption. Following a case of SGA poisoning in year 2015, the German Federal Institute for Risk Assessment (BfR) rather recommends 100 mg kg^−1^ as the upper limit (Bundesinstitut für Riskobewertung (BfR), 2018). It should be noted that the potato tuber also contains other forms of anti-nutritional substances, such as the calystegine alkaloids (CA), a form of polyhydroxylated nortropane alkaloids (Biastoff and Dräger, 2007; Friedman and Levin, 2016; Kohnen-Johannsen and Kayser, 2019). This type of alkaloids is in contrast to the SGAs derived from the polyamine putrescine. Through a series of reactions, initiated by a methylation reaction catalyzed by the enzyme putrescine methyltransferase, putrescine is converted into the key intermediate tropinone. In the potato, tropinone reductase II then initiates a series of reactions leading to the pseudotropine-derived CAs. The CAs are commonly divided into three groups (A, B, and C), depending on their degree of hydroxylation. Although concerns have been raised regarding anti-nutritional effects of CAs, mainly due to their action as glycosidase inhibitors, more research is needed to characterize the toxicity of this group of alkaloids, and recommended safe levels or guidelines are presently lacking (Binaglia et al., 2019).

A few table potato cultivars have been withdrawn from the commercial market after exceeding the recommended SGA limit. High SGA levels were noted in the US cultivar ‘Lenape,’ and this leads to its withdrawal from commercial sales (Sinden and Webb, 1974). A similar situation occurred in Sweden regarding ‘Magnum Bonum,’ a cultivar that ranked third in Swedish production during the early 1980s. The cultivar was in year 1986 shown to contain high SGA levels in several commercial lots (Hellenäs et al., 1995). A small number of consumers showed signs of SGA intoxication and needed to seek medical care. This eventually led to a withdrawal of the cultivar from commercial sales in Sweden. However, the environmental or genetic factors that caused the high SGA levels in Lenape and Magnum Bonum are still not known.

The present study was undertaken to identify genes that could help explain high basal or post-harvest SGA levels in certain potato cultivars such as Lenape and Magnum Bonum. Toward this end, tuber responses to two SGA-inducing treatments, namely, wounding and light exposure, were analyzed using metabolomics and transcriptomics of Magnum Bonum and the reference cultivar Bintje and compared with four additional table potato cultivars of common use in Europe.

## Materials and methods

### Plant materials

Seed tubers from the table potato cultivars Bintje, Juliette, King Edward, and Princess were obtained from certified producers. Desiree and Magnum Bonum seed tubers were generated from *in vitro* clones obtained from Prof. Erland Liljeroth, Swedish University of Agricultural Sciences, Alnarp, Sweden. During years 1 and 2 of the study, tubers were planted in 15-L black plastic pots filled with fertilized peat and grown in an outdoor cultivation facility in Uppsala, Sweden. Tubers were planted at the end of May and harvested after 20 weeks. Watering and fertilization were according to common practice at the facility. All cultivars were grown, harvested, stored, and treated in parallel. Tuber storage after harvest was in a dark room kept at +3°C ( ± 1°C) and at ambient air humidity. Wounding or light exposure treatments of tubers were as described (Petersson et al., 2013a). For all treatments, samples consisted of four tubers of equal size and weight, which were either pooled for metabolic and real-time quantitative polymerase chain reaction (QPCR) analyses or analyzed separately for transcriptomics (n = 3). Samples were frozen in liquid nitrogen and stored at −70°C.

### Generation of transgenic plants

The complementary-DNA fragments (cDNAs) encoding 3-hydroxy-3-methyl-glutaryl-coenzyme A reductase 1 (*HMGR1*) and transaminase isoform 2 (*TAM2*) were reverse transctiptase-PCR (RT-PCR)-amplified from tuber RNA from cv. Magnum Bonum, verified by DNA sequencing, and cloned in sense orientation behind the Cauliflower Mosaic Virus (CaMV) 35S promoter in plasmid pK7WG2.0 (Karimi et al., 2002). As a control, *TAM2* cDNA was also amplified from the EST-clone STMCB25 (Arizona Genomics Initiative, and cloned in sense orientation behind the 35S promoter in plasmid PCV702 (Koncz and Schell, 1986). Plasmids 35S:HMGR1(pK7WG2) and 35S:TAM2(pK7WG2) were used to transform cv. Bintje, whereas 35S:TAM2(PCV702) was used to transform cv. Desiree, as described (Beaujean et al., 1998). Potato transformants were grown in a certified greenhouse with natural daylight supplemented with mercury lamps giving a photon flux of ca. 70 µmol m^−2^ s^−1^, and a 16-h/8-h photoperiod.

### Metabolome analyses

Metabolome fingerprinting was performed using an ultra-high pressure liquid chromatography high-resolution mass spectrometry (UHPLC-HRMS/MS) system consisting of an Acquity UHPLC coupled with Synapt G2 mass spectrometer (Waters, USA). The analytical column was Acquity BEH HILIC (100 mm × 2.1 mm i.d., 1.7 µm; Waters, USA) with column temperature at 40° C. Mobile phases were as follows: A) 5 mM aqueous solution of ammonium formate with 0.1% formic acid (*v/v*) and B) 5 mM solution of ammonium formate in 90% acetonitrile with 0.1% formic acid volume per volume (*v/v*). Gradient elution was as follows: 0-8 min linear gradient elution from 95% to 5% of solvent B, 8% to 12 min isocratic elution at 5% of solvent B, 12–15 min isocratic elution at 95% of solvent B with the flow rate 0.3 mL min^−1^. The High-resolution tandem mass spectrometry (HR-MS/MS) detection used electrospray ionization (ESI) operated in positive and negative ionization modes in the scan range from 100 to 1,200 mass-to-charge ratio (*m/z*). For nebulization nitrogen gas was used. Optimized ESI conditions were as follows: capillary voltage, 1.0 kV in both positive and negative modes; sampling cone voltage, 25 V; extraction cone voltage, 4.0 V; ESI source temperature, 110°C; desolvation temperature, 450°C; cone gas flow, 30 L h^−1^; desolvation gas flow, 800 L h^−1^; collision gas flow 0.5 mL min^−1^; and collision energy ramp in range of 15–45 V. Identification of markers was done on the basis of retention time, exact *m/z* values, elemental composition generated by the MassLynx software (version 4.1), and mass spectra were obtained in full scan HR-MS mode. The compounds were further studied using HR-MS/MS in MS^e^ mode to confirm the identification of compounds according to their fragment ions and MS/MS spectra comparison with databases “Metlin” and “MZcloud.”

### Alkaloid analyses

Glycoalkaloids were from Extrasynthèse (Genay, France). Calystegines A_3_, B_2_, and B_4_ were purified from potato sprouts, and the purity and identity were established by LC-MS and NMR (Schulzova; unpublished work). For all types of alkaloid, the same preparations were used for identification and quantification purposes.

For determination of glycoalkaloids and calystegines, 1 g of freeze-dried sample was shaken with 30 mL of methanol:water (1:1, *v/v*) for 30 min and centrifuged (5 min, 10, 000 revolutions per minute (rpm)). The supernatant was filtered, and the filtrate was quantitatively made up to 50 mL with extractant solution, filtered through a polytetrafluoroethylene (PTFE) microfilter, and diluted 20× before analysis. UHPLC-MS/MS analyses were performed using an UHPLC Dionex UltiMate 3000 RSLC coupled to a Q-Exactive high-resolution tandem mass spectrometer (Thermo Fisher Scientific, Waltham, MA, USA). Chromatographic separation for 2 μL of injected sample extract was carried out using an analytical column Atlantis HILIC Silica (100 mm × 3 mm i.d., 1.7 μm) (Waters, USA) at 30° C column temperature. The mobile phases were A) 20 mM aqueous solution of ammonium acetate and B) acetonitrile, with gradient elution as 0–5 min isocratic elution at 90% of solvent B, 5–8 min of linear gradient elution from 90% to 60% of solvent B, 8–10 min 90% of solvent B with the flow rate 0.45 mL min^−1^.

Mass spectrometric detection was performed using a Q-Exactive high-resolution tandem mass spectrometer equipped with heated ESI, operated in positive ionization modes. The mass spectrometer was operated at a spray voltage of 3.3 kV, heater temperature of 220°C, capillary temperature of 300°C, and S-lens radiofrequency (RF) level of 60. Full MS mode parameters were with a resolution of 70,000 full width at half maximum (FWHM) and scan range of 70−1,000 *m/z*.

Alkaloid identification was performed by comparing the analyte with authentic standards on the basis of retention time and exact mass *m/z*. Quantification of analytes was performed by comparison of the peak area to a standard curve made from serial dilutions of the corresponding reference substance. Analytes and their limit of quantification (LOQ) are given in **Table S2**.

### Transcriptomic analyses

RNA was extracted from leaves and tubers as described (Nahar et al., 2017). Complementary DNA was synthesized using Superscript III reverse transcriptase (Invitrogen Corp., Carlsbad, CA, USA). QPCR was performed with ABsoluteTm QPCR SYBR® Green Fluorescein mix (Thermo Fisher Scientifics Inc., Surrey, UK), analyzed on an iQ5 Real-time PCR detection system (Bio-Rad Laboratories Inc., Hercules, CA, USA), and evaluated using the QBASE software (Hellemans et al., 2007). QPCR was made in triplicates, normalized against two reference genes (60S rRNA protein and β-tubulin), and included an internal reference RNA to standardise for between-run variation. Reference genes were selected from QPCR analyses of six potato genes showing minimal expression changes in a microarray study of wound- and light-inducible genes (Nahar et al., 2017). Primer DNA sequences are listed in **Table S3**.

RNA for transcriptomic analyses was purified from single tubers in biological triplicates. Sequencing libraries, cluster generation, and pair-end sequencing were made by the SNP&SEQ Technology Platform at Uppsala University, following an accredited method using NovaSeq and v1 sequencing chemistry (Illumina Inc.).

Quality control of reads was performed using FastQC algorithm (Andrews, 2010). Reads were aligned against potato genome v.4.03, and transcript counts were estimated using featureCount algorithm in the Subread aligner package (Liao et al., 2013). Transcript coordinates were based on PGSC_DM_V403_representative_genes.gff obtained3from the Solanaceae genome website (http://solanaceae.plantbiology.msu.edu/pgsc_download.shtml). DESeq2 R package was used for differential expression analyses, and a gene was considered as a differentially expressed gene (DEG) when having a fold change (FC) >4.0 and an adjusted p-value <0.01. Transcripts with average values below 20 reads were not included. For better estimates of logfold changes, apeglm shrinkage was performed using lfcShrink function (Zhu et al., 2019).

## Results

### Metabolomic characterization of potato tubers during SGA-inducing conditions

To characterize metabolic responses in potato tubers during SGA-inducing conditions, an untargeted metabolomic analysis was undertaken in tubers from six table potato cultivars that had been subjected to either wounding or light exposure treatment. To also investigate the effect of tuber storage, tubers were analyzed both at harvest and after a cold storage in darkness during 6 months.

**Figure 1:**
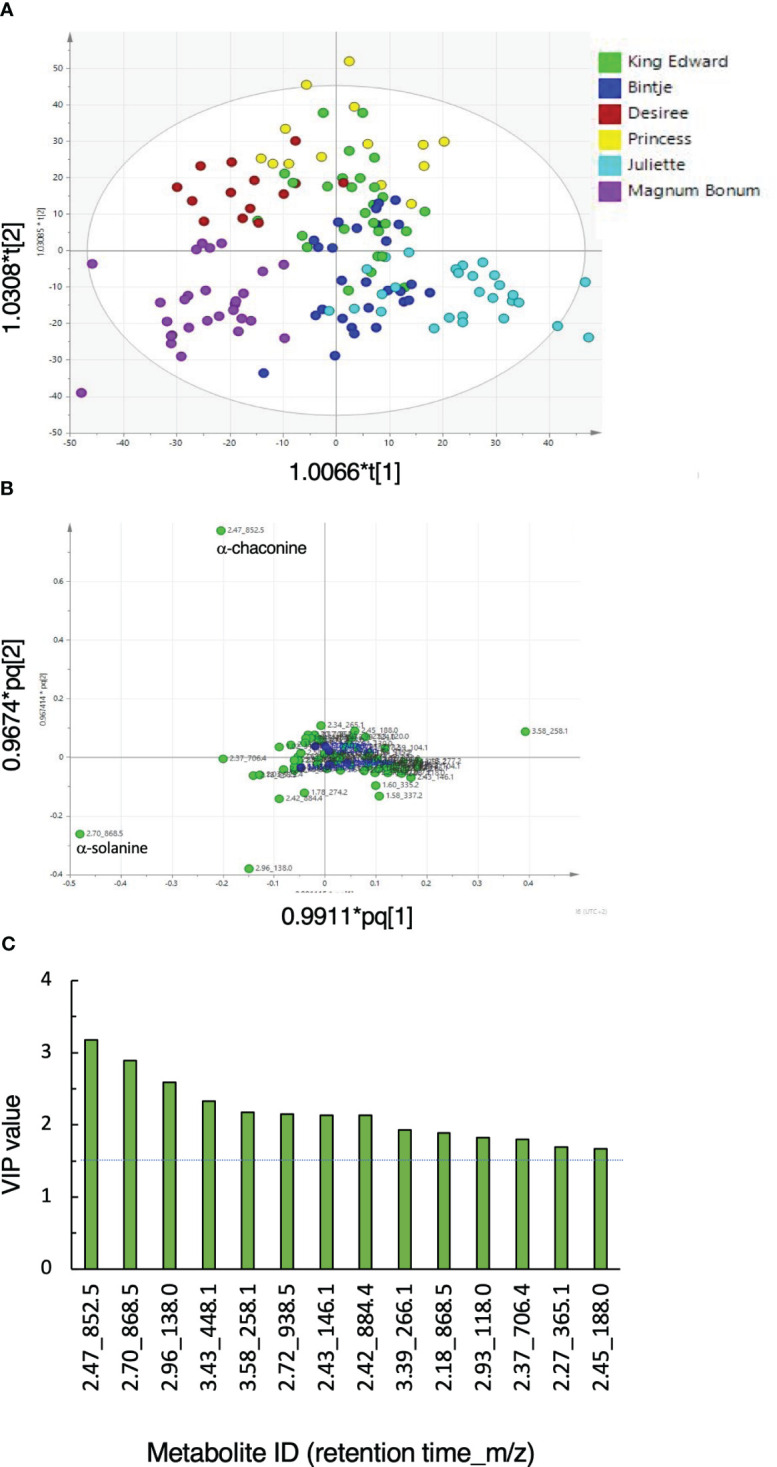
General metabolic responses in potato tubers subjected to wounding or light exposure treatments. Tubers from six potato cultivars were obtained from 1 or 2 years of cultivation, stored or not during 6 months, and treated or not by wounding or light exposure. **(A)** Orthogonal partial least-square discriminant analysis (OPLS-DA) score scatter plot of the first two predictive components of LC-MS metabolite analyses. **(B)** Corresponding loading scatter plot for peak areas (green circles). A set of quality control samples was run in parallel (blue circles). **(C)** Features with variable importance in projection (VIP) values >1.5 (dotted line). Model statistics: R2X = 0.729; R2Y = 0.673; Q2 = 0.555; ellipse indicates Hotelling’s T2 (95%).

The metabolome fingerprint analysis using UHPLC-HRMS/MS acquired a total of 43 features in the negative ionization mode and 73 features using positive ionization. Data acquired in the positive ionization mode were, hence, used for further evaluations. An orthogonal partial least squares discriminant analysis (OPLS-DA) showed cultivar separation on the scores plot (**Figure 1A**). A set of parallel quality control samples grouped near the center of the loading plot, confirming reliable data acquisition (**Figure 1B**). Loading and variable influence on projection (VIP) analyses showed that a number of features, e.g., with the retention time and *m/z* of 2.47_852.5 (α-chaconine), 2.70_868.5 (α-solanine), and 3.58_258.11 (glycerophosphocholine), were important to discriminate between the cultivars (**Figure 1C**). Analyses of the wound and light treatments separately showed that the tuber responses to both treatments were progressive processes (**Figures S2** and **S3**). On the basis of VIP values, a number of features that were important for the discrimination of cultivars in the different OPLS-DA models (cultivar, wound, and light) could be identified, including α-solanine, α-chaconine, and 2.18_868.504 (solamargine) and 2.53_884.499 (solasonine). A summary of response marker metabolites and the corresponding statistical model characteristics is given in **Table S4**.

The results show that glycoalkaloids such as α-chaconine and α-solanine are important parts of metabolic differences between table potato cultivars. This was observed under all conditions investigated, i.e., at harvest, after storage, and after wounding or light exposure.

### Target LC-MS analyses of glycoalkaloids and calystegines

To characterize the tuber alkaloid responses in greater detail, levels of different SGA forms were analyzed by targeted LC-MS analysis of the same materials that had been used for metabolomics. The levels of CAs were analyzed for comparison.

#### Glycoalkaloid quantifications

The quantifiable SGA forms in the investigated cultivars were almost exclusively α-solanine and α-chaconine. Two other SGA forms, namely, solamargine and solasonine, could be quantified in a small fraction (8%) of the analyzed samples, mainly consisting of wounded samples at 48 h from the cultivars Bintje and Desiree. Solasonine and solamargine were identified on the basis of having the same *m/z*, mass spectrum, and retention time in the LC-MS analysis as a corresponding commercial standard, although analyses to fully exclude structurally similar SGA forms, e.g., leptinines, were not done. However, the contribution of these additional glycoalkaloids to total SGA was small, on average 1.7% when quantifyable, the reason why they were not considered in further calculations of total SGA levels. Levels of three other steroidal alkaloids; solanidine, demissidine, and tomatidine, were also assayed but were for all cultivars and treatments below the LOQ.

**Figure 2:**
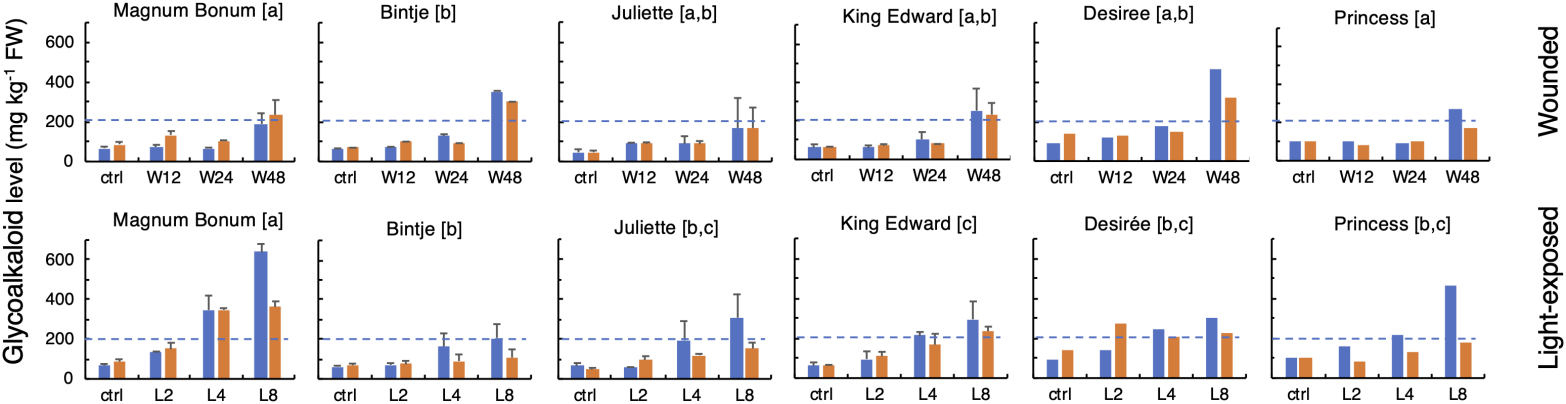
Glycoalkaloid levels in potato tubers subjected to wounding or light exposure treatments. Total glycoalkaloid levels were in a 2-year study analyzed in tubers at time 0 or at different time points after wounding (12 h, 24 h, and 48 h; upper) or white light exposure (2 days, 4 days, and 8 days; lower), either at harvest (blue bars) or after a cold storage for 6 months in darkness (yellow bars). Mean level of total glycoalkaloids ± range for 2 years (MB, Bi, Ju, and KE) or year 1 only (De and Pr). Different letters in brackets indicate statistically different responses (p < 0.05, ANOVA). Dotted lines indicate the recommended upper safe level of 200 mg total SGA kg^−1^ FW in raw unpeeled tubers.

There were no significant differences between the cultivars for their total SGA level in the control (i.e., untreated) samples as measured at harvest or after the 6-month storage (**Figure 2**). As expected, both wounding and light exposure treatments of tubers increased total SGA levels significantly. A statistically significant SGA increase compared with untreated controls became detectable at 24 h after wounding and at 48 h after light exposure (p < 0.05, *t*-test). This was true both at harvest and after the six-month storage period. There was no significant correlation between the SGA increases following wounding and light exposure treatments (not shown), well in line with a previous investigation (Petersson et al., 2013a).

The tuber SGA level after wounding or light exposure treatment differed significantly between the cultivars (**Figure 2**). The highest final levels after wounding were observed for the cultivars Bintje and Desirée, which displayed SGA levels over 300 mg kg^−1^ (FW), significantly higher than those of the other four cultivars. The SGA response after a light exposure showed a greater between-cultivar variation. The strongest response was in this case observed in Magnum Bonum, and the levels above or close to 600 mg kg^−1^ were during both cultivation years detected after 8 days. In addition, cv. Princess displayed a relatively high SGA level after light exposure (464 mg kg^−1^). The lowest degree of light induction among the six cultivars was detected in Bintje, which, after 8 days of light exposure, barely reached the level of 200 mg kg^−1^.

To verify these apparent cultivar differences in light responsiveness, the light exposure experiment was as a control repeated 5 years later, specifically for the cultivars Bintje, Magnum Bonum, and Princess. This showed essentially the same SGA levels and between-cultivar variation as in the former analyses (**Table 2**). Thus, for 3 years of cultivation and SGA analyses spanning a total period of 7 years, Magnum Bonum consistently displayed about three times higher SGA levels after 8 days of light exposure than Bintje, on average, 629 mg kg^−1^ vs. 214 mg kg^−1^, whereas their control levels at harvest were close to identical (**Table 2**). A light-induced SGA increase was thus significantly higher in Magnum Bonum than in Bintje (p < 0.001, *t*-test). The light response in Princess was intermediate, on average 452 mg kg^−1^, but still well above the recommended safe limit of 200 mg kg^−1^.

**Table 2:** SGA levels in potato tubers at harvest and after light exposure.

Year	Bintje		Princess SGA (mg kg^−1^ FW)		Magnum Bonum	
	Harvest	Light	Harvest	Light	Harvest	Light
Year 1	55	138	105	464	60	680
Year 2	67	277	n.d.^b^	n.d.	75	596
Year 7	54	226	31	440	61	610
Mean ± SD	59 ± 6	214 ± 57	68 ± 37	452 ± 12	65 ± 7	629 ± 37
Fold change^a^		3.6		6.7		9.7
*t*-test vs. Bintje			n.s.^c^	p <0.05	n.s.	p <0.001

Potato tubers originating from outdoor plant cultivations in 15-L pots were during 3 separate cultivation years analyzed at harvest and after 8 days exposure to white fluorescent light (100 µmol m^−2^ s^−1^). Total SGA levels were calculated from the sum of α-solanine and α-chaconine as analyzed by LC-MS.

^a^Fold change relative to the SGA level at harvest. ^b^Not done. ^c^Not significant.

Visual inspection of tubers after light exposure showed a variation of skin colors (**Figure S4**). There was no obvious correlation between the change of skin color and SGA alterations. For instance, Magnum Bonum displayed the highest light-induced SGA increase but attained only a weakly brownish skin color alteration. By contrast, Desiree attained a strongly red/blue skin color but had only a moderate SGA increase. Skin color changes could thus be related to SGA levels only for the individual cultivar (Grunenfelder et al., 2006).

Cold storage of tubers during 6 months did not alter SGA levels significantly in the untreated control samples compared with the measurements made at harvest (**Figure 2**). The storage period seemed to attenuate the wounding response to some extent and led to a significantly weaker SGA response to light exposure at 8 days (p < 0.05, paired *t*-test). However, the relative order between cultivars in light-treated tubers did not change after storage, e.g., Magnum Bonum still produced the highest SGA levels and Bintje the least.

#### Calystegine alkaloid quantifications

As a metabolic point of comparison, the levels of the tropane-derived CAs were determined in the same samples as those used for SGA analyses. The main CA form in all genotypes was CA_A3_, which typically accounted for 75% or more of total CA levels. Additional CA forms detected were CA_B2_ and CA_B4_. Thus, total CA levels were calculated from the sum of CA_A3_, CA_B2_, and CA_B4_.

The highest CA levels were detected in cvs. Desiree and Magnum Bonum, whereas the lowest levels were seen in Princess, the range being almost 20-fold (**Figure S5**). CA levels at tuber harvest were for all cultivars not different from the CA level after 6-month storage. Furthermore, CA levels did not change after wound or light exposure; the latter in contrast to the parallel SGA increases. There was thus no significant correlation between CA and SGA levels for any cultivar. Genotype differences were thus more important than treatment ones, with respect to CA levels.

### Transcriptomic analyses

To investigate the genetic basis for the cultivar differences in SGA production, we performed an RNAseq analysis of the cultivars Bintje and Magnum Bonum, i.e., two cultivars that showed significant SGA response differences in tubers subjected to wounding or light exposure. The analyzed tuber samples corresponded to those used for SGA analyses during year 1 of the study. Because the expression of genes in SGA metabolism precedes the SGA accumulation (Nahar et al., 2017), the RNAseq analysis was performed before the end-point SGA measurements, i.e., at 24 h after wounding or at 48 h after light exposure.

Principal component analysis (PCA) of gene expression patterns showed that samples clustered within the treatment (**Figure S6**). A certain separation of the cultivars was evident for the light exposure treatment, in line with the observed SGA differences. Depending on the cultivar and treatment, there were in total between 2,500 and 3,500 DEGs (**Figure S7A**). Venn display of DEGs revealed 287 genes whose expression in the two cultivars were significantly upregulated during both wound and light treatments and 179 DEGs that were downregulated (**Figures S7B, C**; **Tables S5** and **S6**).

Genes accounting for significant response differences were determined from analyses of gene × cultivar interactions in a multivariate linear regression model, and their different expression patterns were illustrated by Volcano and Heatmap displays (**Figure S8**). Analyses of the 100 most significant gene × cultivar interactions showed a cultivar separation for both wounding and light treatments (**Figure S8**; **Tables S7** and **S8**), but none of the genes could be attributed to a role in sterol or alkaloid metabolism. Rather, they seemed to be part of cultivar differences related to other types of wound- or light-regulated processes. Gene ontology (GO) analysis further illustrated this and showed that genes associated with the photosynthetic metabolism were overrepresented in Bintje during both treatments (**Figure S9**). In Magnum Bonum, the regulation of metabolic and oxidative processes was overrepresented in wounded and light-exposed tubers, respectively.

**Figure 3:**
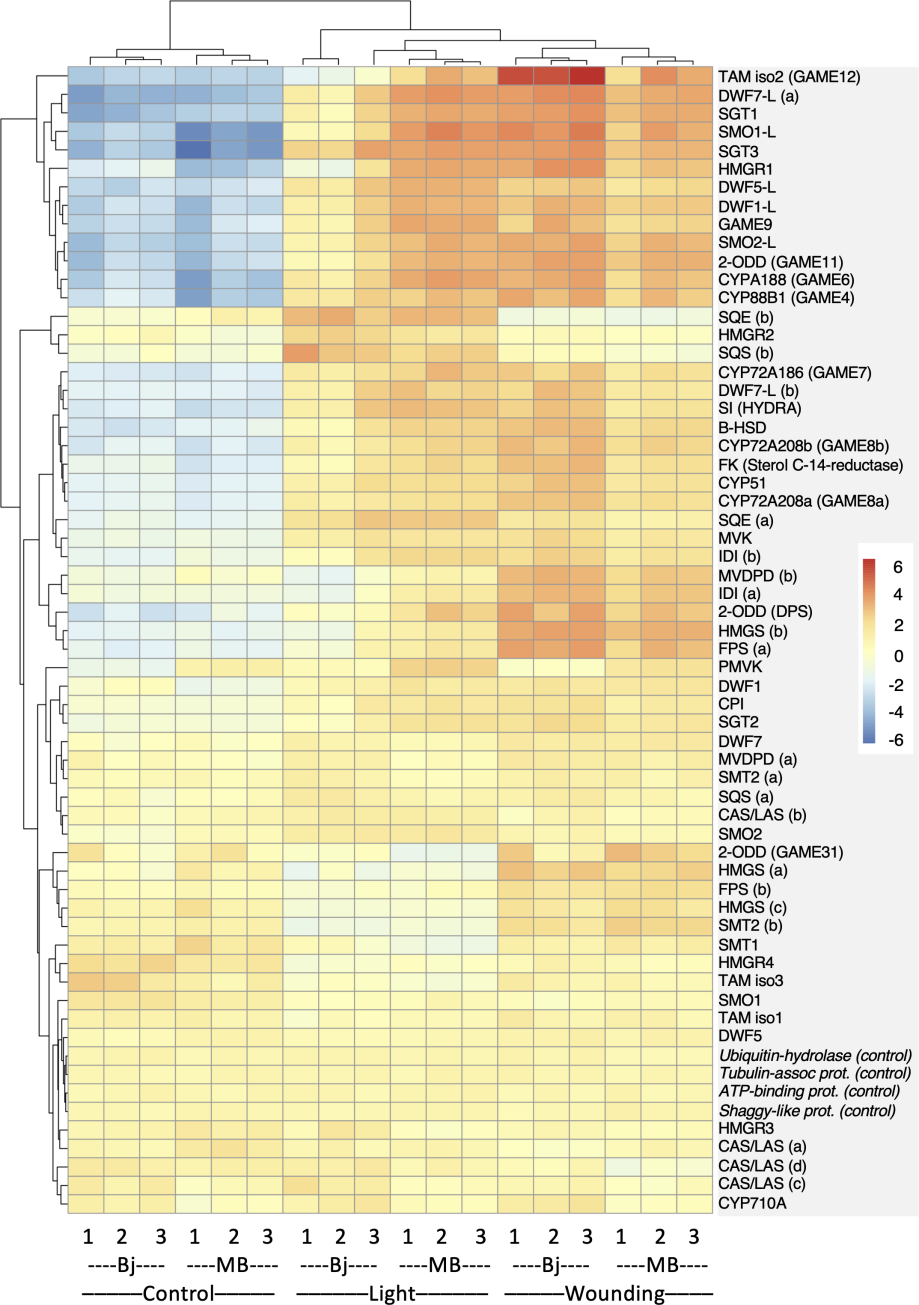
Cultivar differences in sterol- and SGA-related gene expression in Bintje (Bj) and Magnum Bonum (MB) potato tubers subjected to wounding or light exposure treatments or not (control). Expression of 58 selected genes with a role in sterol and SGA synthesis was monitored in a RNAseq study with triplicate samples. Four genes not regulated by wound or light were included as controls. Colors indicate variance stabilizing-normalized gene expression values after mean subtraction. Gene annotations and expression data are given in **Table S9**.

To more directly pinpoint the genes that might underlie the observed cultivar differences in SGA accumulation, we specifically monitored the expression of 58 genes encoding proteins with a known or plausible role in sterol- and SGA biosynthesis. Heatmap display of this gene set indicated a signficant shift in the expression profile of a number of genes after both wounding and light exposure (**Figure 3**; **Table S9**). Cluster analysis of gene expression profiles identified three clusters (#1, #2, and #3) that were positively correlated with increased SGA levels (**Figure S10**). These clusters included not only genes encoding sterol-biosynthetic enzymes such as HMGR1, SMO1-L, SMO2-L, and DWF7-L, as well as proteins acting in the post-cholesterol SGA metabolism, e.g., CYP72A188, 2-ODD (GAME11), TAMisoform2 (TAM2 and GAME12), SGT1, and SGT3, but also the SGA-related transcription factor GAME9. These results correlated well with the wounding and light induction of *HMGR1*, *SMO1-L*, *CYP72A188*, *GAME11*, *TAM2*, *SGT1*, and *SGT3*, as observed in a potato microarray study (Nahar et al., 2017). Interestingly, the transcription factor GAME9 grouped in the same cluster (#3) as some of its supposed target genes in potato, e.g., *DWF1-L*, *DWF5-L*, and *GAME11* (Cárdenas et al., 2016), indicating that the gene in itself was inducible by both wounding and light exposure. This was confirmed by QPCR analyses, showing not only significant wound and light inductions of *GAME9* expression in Bintje and Magnum Bonum during both years of the study, but also that the degree of induction did not differ between the cultivars (**Figure S11**).

In light of recent work in tomato showing two allelic GAME9 variants with different affinities for their target genes *GAME17* and *GAME7*, i.e., GAME9^135V^ (low) and GAME9^135A^ (high) (Yu et al., 2020), we analyzed the full-length GAME9 cDNA sequence in Bintje and Magnum Bonum. This showed the GAME9^135A^ variant in both cultivars and that the deduced GAME9 amino acid sequences were identical over the entire protein (**Figure S12**). The GAME9^135A^ form was present also in two other potato cultivars for which corresponding DNA sequences were available, as well as the wild potato species *S. Phureja*.

**Figure 4:**
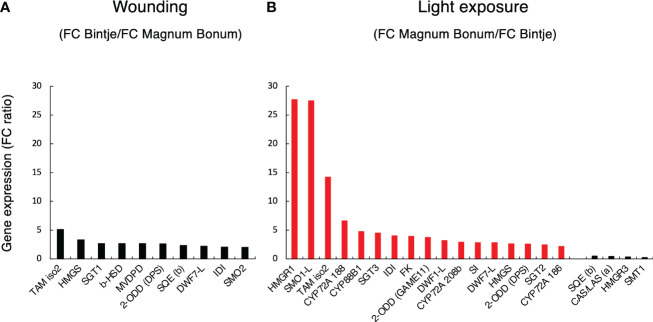
Cultivar differences in sterol- and SGA-related gene expression in potato tubers subjected to wounding or light exposure treatments. Expression of 58 genes with a known or plausible role in sterol and SGA synthesis was monitored in a RNAseq study. Subsequent data filtering was based on a statistically significant gene × cultivar interaction for the treatment (p < 0.05) and a >2-fold gene expression fold change (FC) for the cultivar. **(A)** Between-cultivar FC ratio of filtered genes in Bintje relative to Magnum Bonum after wounding. **(B)** Between-cultivar FC ratio of filtered genes in Magnum Bonum relative to Bintje after light exposure. Corresponding end-point SGA levels were higher in Bintje after wounding and higher in Magnum Bonum after light exposure (**Figure 2**). Black and red bars indicate a higher FC in Bintje and Magnum Bonum, respectively.

To further illustrate the most important gene expression differences between the two cultivars, the set of 58 sterol- and SGA-related genes was filtered both for a significant gene × cultivar interaction (P_adj_ < 0.05) and an over two-fold treatment difference between cultivars. This identified 10 genes after wounding and 21 genes after light exposure (**Figure 4**). The span of between-genotype FC ratios was smaller after wounding than after light exposure, in line with the observed smaller SGA differences between genotypes after wounding than after light treatment. The largest FC difference between the cultivars after wounding was a five-fold higher *TAM2* (*GAME12*) induction in Bintje than in Magnum Bonum (**Figure 4A**). In the light experiment, the span of FC-ratios was greater than after wounding. In particular, three genes —*HMGR1*, *SMO1-L*, and *TAM2* —showed much higher FC values in Magnum Bonum than in Bintje (**Figure 4B**). In addition, downregulation of four genes —*SMT1*, *SQE*, *HMGR3*, and *CAS—* was significantly stronger in Magnum Bonum after light exposure.

#### Validation of transcriptomic analyses

To validate the transcriptomic analyses, relative expression levels of *HMGR1*, *SMO1-L*, *TAM2*, and *SMT1*, as well as of six additional sterol- and SGA-related genes, were monitored by QPCR in tubers from all six cultivars. With respect to Bintje and Magnum Bonum, this showed for year 1 a good agreement between the QPCR values and the equivalent gene expression levels determined by RNAseq of the same samples (**Figure S13A**). Furthermore, a comparison of all six cultivar´s QPCR values from year 1, with the equivalent ones from year 2, showed that their gene expression levels were well correlated between both cultivation years (**Figure S13B**). This agreed with the relatively small SGA differences between the 2 years (**Figure 2**). The null hypothesis, i.e., no correlation between the factors analyzed, could be rejected for both these comparisons (p < 0.0001; *t*-test).

**Figure 5:**
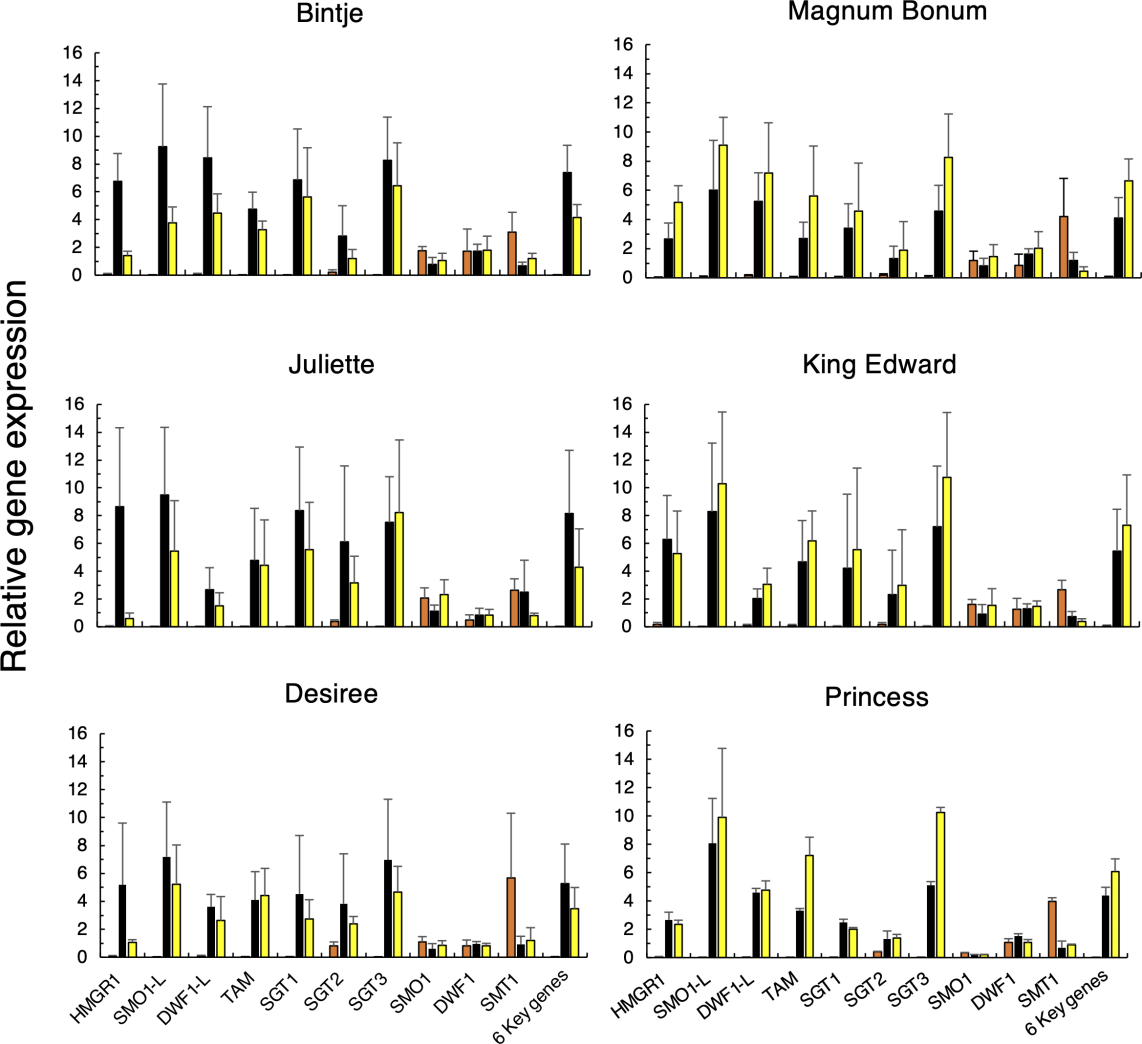
Relative gene expression of ten SGA-related genes in potato tubers subjected to wounding or light exposure treatments. Tubers were obtained from 2 separate years of cultivation (only year 1 for Princess), stored or not for 6 months, and treated or not by wounding (24 h) or light exposure (48 h, white fluorescent light; 100 µmol m^−2^ s^−1^). Gene expression was analyzed in technical triplicates by QPCR in pooled cross sections from four tubers and then normalized using two genes as internal reference. Average values ± SD from n = 4 separate analyses during 2 years for each gene and treatment (n = 2 analyses for Princess) for control (orange bars), wounded (black bars), and light-exposed tubers (yellow bars). Six key genes indicate the average of the six genes with the strongest treatment induction. Gene abbreviations are given in **Table 1**.

The QPCR analyses also showed that the relative wound- vs. light-regulated expression of the stress-inducible SGA genes (*HMGR1*, *SMO1-L*, *DWF1-L*, *SGT1*, *SGT3*, and *TAM2*) differed among the six cultivars (**Figure 5**). The wound induction of these genes in Bintje was stronger than their induction by light, whereas the opposite was true for Magnum Bonum (p < 0.001, paired *t*-test); also this being in agreement with the corresponding SGA differences (**Figure 2**). The other four cultivars had expression profiles that were either more similar to Bintje (Juliette, Desiree) or to Magnum Bonum (Princess, King Edward). The QPCR results also showed that the downregulation of *SMT1* and *SMO1* by wounding or light exposure, as observed in the transcriptome analyses of Bintje and Magnum Bonum, was true for all six cultivars investigated.

For all cultivars analyzed during 2 years, a linear regression of the relative gene expression vs. the final SGA level showed a positive and significant correlation for seven genes: *HMGR1*, *TAM2*, *DWF1-L*, *SMO1-L*, *SGT3*, *SGT1*, and *SGT2* (**Table 3**). Furthermore, a significantly negative correlation was observed for *SMT1* and *SMO1* expression, whereas the null hypothesis could not be rejected for *DWF1*.

**Table 3:** Correlation between relative gene expression and SGA levels in potato tubers subjected to two SGA-inducing treatments.

Gene	Slope (*mg SGA qpcr expr^−1^*)	*t*-test
Positive correlation vs. SGA		
*TAMiso2*	141	p < 0.0001
*DWF1-L*	137	p < 0.0001
*HMGR1*	134	p < 0.0001
*SGT3*	88	p < 0.0001
*SMO1-L*	79	p < 0.0001
*SGT2*	99	p < 0.01
*SGT1*	68	p < 0.01
Negative correlation vs. SGA		
*SMT1*	−101	p < 0.05
*SMO1*	−256	p < 0.05
*No correlation vs. SGA* *DWF1*		n.s.^a^

Expression of ten genes with a role in sterol/SGA synthesis was monitored by QPCR in tubers from five potato cultivars that had been subjected to a wounding (24 h) or a light exposure (48 h) treatment either at tuber harvest or after 6 months of tuber storage under dark and cold conditions. Tuber SGA levels were quantified by LC-MS in the same experiments, either after 48 h (wounding) or 8 days (light). The experiment was performed for 2 cultivation years, giving in total 56 data pairs for each gene. A correlation between gene expression and SGA level was analyzed by linear regression of untransformed data, and a t-test of the slope difference from zero.

a Slopes with p-values >0.05 were considered as being not significantly different from zero (n.s.).

Together these QPCR analyses corroborate the transcriptomic analysis from year 1 of the study and show that the effect of wounding or light exposure treatments on gene expression was well correlated between the 2 years.

### Overexpression of key SGA genes in transgenic potato plants

The significantly stronger light-induced expression of certain SGA genes, such as *HMGR1*, *SMO1-L*, and *TAM2* in Magnum Bonum, might explain the cultivar’s higher SGA levels after light exposure. To investigate a role of these genes in SGA production, we generated transgenic Bintje plants overexpressing the Magnum Bonum *StHMGR1_MB_* and *StTAM2_MB_* cDNAs from the 35S promoter. As a control, transgenic Desiree plants were created expressing an independent *StTAM2_Ken_* cDNA clone from cv. Kennebec.

**Figure 6:**
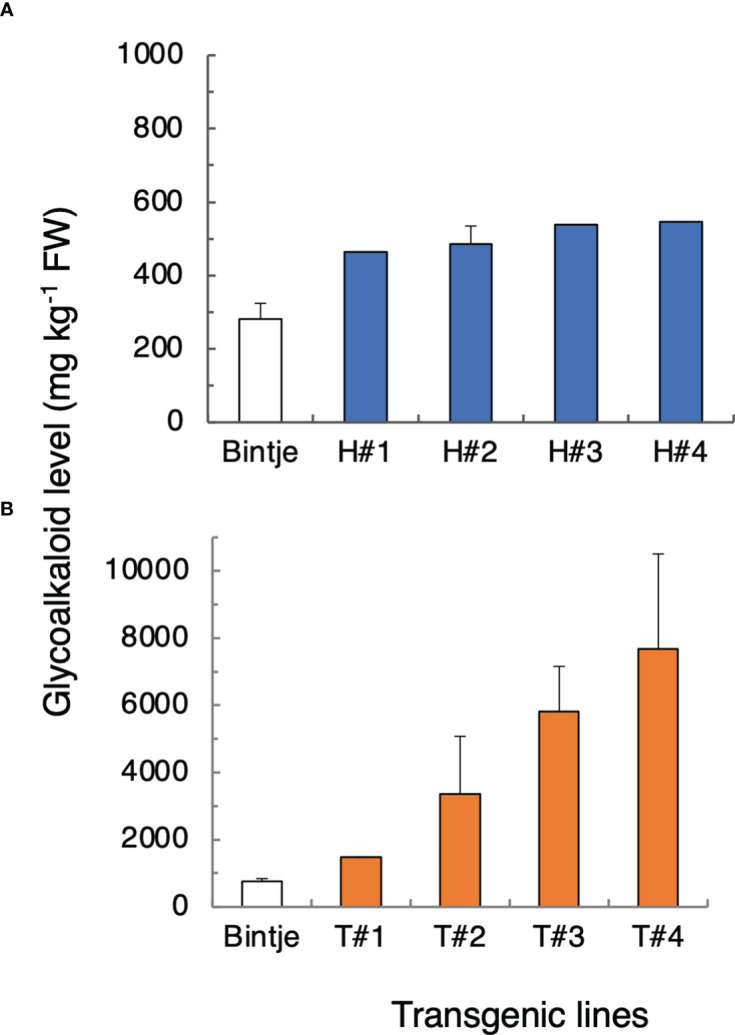
Total glycoalkaloid (SGA) levels in leaves from transgenic potato plants overexpressing *StHMGR1_MB_* (H) and *StTAM2_MB_* (T) cDNAs. **(A)** Mean SGA level ± SD or range, from the sum of α-chaconine and α-solanine as measured by LC/MS, in wild-type Bintje plants (n = 4 plants; white bars) and 35S:*StHMGR1* transformants (n = 1 or 2 plants per line; blue bars). **(B)** Mean SGA level in wild-type Bintje (n = 6 plants; white bars) and 35S:*StTAM2_MB_* (n = 2 or 3 plants per line; yellow bars). Plants in **(A, B)** were grown at separate occasions. For both types of transformant, lines with four highest SGA levels among at least eight independent lines analyzed are shown. A statistical difference of SGA levels between the transformants and the wild type was significant at p < 0.001 (Student´s *t*-test). Corresponding transgene expression values are depicted in **Figure S14**.

Compared with control plants, Bintje 35S:*StHMGR1_MB_* lines accumulated up to about 100% more SGA in leaves, whereas 35S:*StTAM2_MB_* transformants accumulated up to 900% more, the increases being statistically significant for both types of transformant (**Figure 6**). There was a variation of SGA levels within the transgenic populations (not shown), presumably related to the degree of transgene expression. QPCR analyses showed that both the *StHMGR1_MB_* and *StTAM2_MB_* transgene expressions were correlated to SGA levels (**Figure S14**). In line with SGA increases in 35S:*StTAM2_MB_* transformants, overexpression in Desiree of the *StTAM2_Ken_* cDNA also led to significant leaf SGA increases, but, in this case, up to 100% more (**Figure S15**). The different relative SGA increases in Bintje vs. Desiree *StTAM2* transformants may be due to the use of different cDNAs, vectors, or cultivars. Further analyses of SGA levels in the corresponding Bintje transgenic tubers revealed only insignificant SGA alterations compared with controls (not shown), possibly due to the tissue-specific expression differences between the 35S promoter activity in the tuber flesh (Kim et al., 2009) and the localization of the general SGA synthesis machinery in tuber phelloderm (Krits et al., 2007).

The results show that an increased expression of *StHMGR1*or *StTAM2* alone is sufficient to increase SGA levels. Enzymatic activity of the encoded proteins can thus be seen as being limiting for SGA biosynthesis.

## Discussion

### Glycoalkaloid and calystegine alkaloid variation in table potato cultivars

Metabolomic profilings showed that α-solanine and α-chaconine were important parts of the general metabolic differences that existed between the six potato cultivars that were investigated. This was shown for tubers analyzed directly at harvest and after wounding or light exposure (**Figure 1**). In addition, CA levels varied between the cultivars but were not significantly altered by the SGA-inducing treatments (**Figure S5**). These findings agree well with previous studies (Petersson et al., 2013a; Shepherd et al., 2016) and demonstrate that increased SGA levels do not occur at the expense of CA biosynthesis, not even during the 10-fold SGA increases that were here observed in light-induced Magnum Bonum tubers. The SGA and CA biosynthesis pathways can thus be seen as two metabolically independent routes for alkaloid production in the potato. In a food quality perspective, analyses of both SGA and CA are thus needed to evaluate the tuber quality at harvest, but additional analyses mainly of SGA might be sufficient to monitor later post-harvest changes. The negligible levels of additional SGAs in all samples shows that α-solanine and α-chaconine are the most important SGAs to monitor in table potato tubers, also when stresses and post-harvest conditions are to be considered.

We have previously reported that the relative degree of SGA increases in tubers after wounding or light exposure differs between cultivars (Petersson et al., 2013a). We here confirm and extend these findings and show that cultivar differences in tuber SGA accumulation after both wounding and light exposure are stable for a time period of at least 2 and 7 years, respectively. This strongly suggests that the between-cultivar differences in SGA accumulation are genetically determined. Results also suggest that the relative degree of wound or light responsiveness are separable genetic traits.

Cold storage of tubers in the dark for 6 months did not significantly alter SGA levels in control tubers, although a certain reduction in both wound and light responsiveness was observed. A similar decrease in light responsiveness after cold storage has been shown also for the cultivars Kennebec and Russet Burbank (Mondy and Seetharaman, 1990). These results may reflect a reduction of tuber viability during storage but without affecting the original SGA level attained at harvest. However, also stronger light inductions have been reported after tuber storage (Percival et al., 1993; Griffiths et al., 1998). The reason for these contrasting results is not known, and the effect of storage on tuber SGA responses is seemingly a subject that merits more investigation.

### Magnum Bonum tubers have an unusual strong responsiveness to light exposure

The strongest SGA increases in the present study were detected after light exposure of cv. Magnum Bonum, where 8-day light treatments during 3 separate cultivation years increased the SGA levels about 10-fold compared with the harvest level (**Table 2**). These increases were significantly higher than those observed for any of the other five cultivars investigated in parallel. Investigations using comparable experimental conditions have shown between two-fold and five-fold SGA inductions for the over 30 potato cultivars analyzed, the average being 2.4-fold (Percival, 1999; Chuda et al., 2004; Petersson et al., 2013a). This inidicates that the degree of light induction of SGA in Magnum Bonum is unusually high among table potato cultivars. By contrast, the relative SGA induction in Magnum Bonum after wounding was not stronger than for any of the five additional cultivars that we investigated here or among the 21 cultivars investigated by Petersson et al. (2013a). In fact, the wounding response of Magnum Bonum was in the present study significantly lower than that of Bintje (**Figure 2**). Together, the results show that Magnum Bonum compared with most table potatoes has a relatively normal tuber SGA level both at harvest and after a wounding treatment, but that its SGA level can become extremely high as a response to light exposure.

Magnum Bonum was withdrawn from commerce in Sweden after having demonstrated high SGA levels after a cold and wet autumn in year 1986 (Hellenäs et al., 1995). SGA levels reported from analyses of tubers from over 300 commercial lots ranged between 61 and 665 mg kg^−1^, the mean level being 251 ± 106 mg kg^−1^. The range of SGA levels for light-exposed Magnum Bonum tubers observed in our present study, between 60 and 680 mg kg^−1^, is thus remarkably similar to that described by Hellenäs et al. (1995). As analyzed in a questionnaire to the Magnum Bonum farmers in 1986, both frost and mechanical vine killing showed significant correlations to high SGA levels (Diedrichs, 1988), but a potentially causal relationship was not further investigated.

We suggest that the sudden increase of high SGA levels in Magnum Bonum tubers at least, in part, can be explained by the cultivar’s unusual strong light responsiveness. The SGA induction in potato tubers subjected to light exposure is triggered already at low light intensities, e.g., 7–14 µmol m^−2^ s^−1^ (Grunenfelder et al., 2006; Olsen et al., 2018), and natural daylight has a spectral composition that is active in the SGA induction (Sinden et al., 1984; Percival et al., 1996). We speculate that an increased soil erosion around tubers caused by the heavy raining or mechanical vine killing, possibly in combination with inadequate hilling, may have led to an exposure of tubers to low levels of daylight that was sufficient to trigger a high SGA synthesis in this light-sensitive cultivar. In support of this view is that potatoes that are planted with a small soil cover and not are hilled during the growing season form higher SGA levels than those that are planted deeply and kept well covered (Hutchinson and Hilton, 1955). Similarly, covering the soil with black film during the growth season lowered SGA levels in tubers at harvest with over 50%, depending on the cultivar (Nie et al., 2021). In addition, in line with our hypothesis is that the range of Magnum Bonum SGA levels detected in year 1986 compared well to those we here obtained by light exposure but were clearly different to those obtained by wounding (**Figure 2**). The greening response of Magnum Bonum tubers after light exposure was weak (**Figure S4**), the reason why a low light exposure of tubers might have been undetected by visual observation at harvest but still could have been able to increase SGA production.

However, other explanations, such as low-temperature stress (van Swaaij, 1992) and/or flooding/anoxia responses (Hellenäs et al., 1995), are also plausible. Interestingly, trancriptome analysis of tubers submerged in water (i.e., “flooded”) did not reveal any significant upregulations of SGA-associated genes (Peivastegan et al., 2019). Instead, the main SGA-related effect was a downregulation of *SGT1* and *SGT3* genes. Because the downregulation of *SGT1* and *SGT3* decreases SGA levels in transgenic plants (McCue et al., 2005; McCue et al., 2007), the results in the work of Peivastegan et al. (2019) indicate lowered SGA levels during flooding, rather than increased. The influence of low temperature or frost remains to be investigated.

Whatever the exact cause of high SGA levels in Magnum Bonum was in year 1986, our results demonstrate that Magnum Bonum has the strongest light responsiveness with respect to the SGA accumulation among more than 30 table potato cultivars that have investigated by us and by others. To what extent a strong light responsiveness also applies for other high-SGA cultivars, such as Lenape, is not known. Our results should stimulate further analyses of the influence of light and/or other conditions on SGA levels in table potato cultivars, as well as breeding lines, to identify the most sensitive genotypes for each condition.

### Key genes associated with high SGA production

Transcriptomic analyses identified a small number of SGA-associated key genes that were more strongly and significantly induced by light in Magnum Bonum than in Bintje. The most significant examples were *HMGR1*, *SMO1-L*, and *TAM2*, but 14 other genes were also shown to be important (**Figure 4**). In addition, four genes were identified whose expression was negatively correlated to SGA levels, *SMT1* being the most significant gene in this case. At present, we do not know what regulatory processes that might underly these cultivar differences in gene expression. The spectral response for the light induction of SGA levels in cv. King Edward potato tubers is consistent with a role for both cryptochrome (blue light) and phytochrome (red light) photoreceptors, similar to the parallel light induction of chlorophyll biosynthesis genes (Okamoto et al., 2020). In addition, in line with a role for the light-regulated gene expression machinery is that downregulation in tomato plants of the light signal transduction factors ELONGATED HYPOCOTYL 5 (SlHY5) and PHYTOCHROME-INTERACTING FACTOR 3 (SlPIF3) modulated expression of SGA biosynthetic genes and reduced SGA levels (Wang et al., 2018). Furthermore, a comparison of the SGA-associated genes in **Figure 4** with those that have been shown to be correlated to the expression of the SGA transcription factor GAME9 in *GAME 9*-overexpressing potato transformants (Cárdenas et al., 2016) showed both similarities and differences. For instance, expression *GAME11* (*ODD*) and *GAME12* (*TAM2*) were elevated in potato *GAME9*-overexpressing transformants, but *HMGR1* or *SMO1-L* was not reported as upregulated. It should, however, be noted that different tissues (leafs vs. tubers) were used in these two studies. We show that the *GAME9* expression in tubers in itself is upregulated by wounding as well as light exposure, both in Bintje and Magnum Bonum (**Figure S11**). However, the degree of wound- or light-induced *GAME9* expression did not differ significantly between the cultivars, and their GAME9 amino acid composition was identical (**Figure S12**), together indicating that GAME9-regulated expression or activity was not main parts of the between-cultivar SGA differences in our investigation.

The increased SGA levels in leafs from transgenic potato plants overexpressing *StHMGR1* and *StTAM2* indicate that induced expression of one single key SGA gene can be sufficient to increase SGA synthesis. The increased leaf SGA levels in our *HMGR1*-overexpessing transformants are well in agreement with other reports on HMGR1-overexpressing potatoes (Ginzberg et al., 2012; Moehninsi et al., 2020; Villano et al., 2020) and suggest a limiting function of the enzyme. The effect of TAM2 overexpression on SGA levels in table potatoes has not been investigated directly, although Nakayazu et al. (2021) showed that overexpression of the potato *PGA4* cDNA (GAME12 and TAM2) could restore SGA production in hairy roots obtained from *Solanum stipuloideum*, a wild potato species lacking SGA due to a natural mutation in the *PGA4* gene. Conversely, we have shown that overexpression of a soybean *SMT1* cDNA in transgenic potato plants reduces SGA levels due to a decreased production of the SGA precursor cholesterol and increased levels of the non-precursors campesterol, sitosterol, and stigmasterol (Arnqvist et al., 2003). The downregulation of SMT1 expression by light exposure in all cultivars here investigated might thus be anticipated as having the opposite effect, i.e., increasing cholesterol synthesis. Well in line with this is that Arabidopsis SMT1 mutants have increased cholesterol levels (Diener et al., 2000) and that cholesterol levels increase during both wound- and light-induced SGA accumulation in potato tubers (Nahar et al., 2017). The role in SGA synthesis for the other three genes (*SQE*, *HMGR3*, and *CAS/LAS*) that were negatively correlated to SGA after light exposure is not clear, but also the expression profiles of these genes can be interpreted in terms of increasing cholesterol production through a reduced synthesis of competing steroidal substances.

These results are consistent with a situation where differences in SGA levels between potato cultivars depend on their individual expression or activity of genes/enzymes that act at key steps in both the pre- and post-cholesterol SGA metabolism, as well as on their activity of genes/enzymes regulating competing steroidal reactions. A model for the main SGA-biosynthetic differences between light-exposed Bintje and Magnum Bonum tubers is depicted in **Figure S16**.

## Conclusion

Collectively, our findings show that a between-cultivar variation in the tuber responsiveness to light exposure is a trait that has been neglected in current attempts to mitigate the SGA hazard in potato. Consequently, it may now be useful to monitor the degree of light responsiveness of both table potato cultivars and of breeding lines. We propose that quantitative expression analyses of key SGA genes (e.g., *HMGR1*, *DWF1-L*, and *TAM2*) can be used as a molecular tool in such screens to simplify identification of cultivars at risk of forming high SGA levels after light exposure, as well as after other SGA-increasing conditions.

## Data availability statement

The datasets presented in this study can be found in online repositories. The names of the repository/repositories and accession number(s) can be found below: European Nucleotide Archive, PRJEB63328.

## Author contributions

IM, AG, VS, and FS conceived and designed the research. IM, AG, UA, EW, and FS performed the plant experiments. AG, AK, JH, VS, and FS performed the metabolomics and statistical analyses. IM, AA, and FS performed the transcriptomics and statistical analyses. IM, UA, SKJ, and FS performed plant transformations and analyzed the transformants. IM, AG, AA, VS, and FS analyzed the raw data. FS wrote the manuscript with input from authors. IM and AG contributed equally. All authors read and approved the final version.
