# Supplementary material for: Metabolomic and transcriptomic analyses identify external conditions and key genes underlying high levels of toxic glycoalkaloids in tubers of stress-sensitive potato cultivars
Source: Front Plant Sci. 2023 Oct 4;14:1210850. doi: 10.3389/fpls.2023.1210850 (PMC10582707; doi:10.3389/fpls.2023.1210850)
Supplement: Supplementary file 2 [file DataSheet_2.pdf]

Supplementary Material

Metabolomic and Transcriptomic Analyses Identify External Conditions and Key Genes Underlying High Levels of Toxic Glycoalkaloids in Tubers of Stress-sensitive Potato Cultivars

Authors: Irene Merino[#], Alexandra Olarte Guasca[#], Ales Krmela, Usman Arif, Ashfaq Ali, Erik Westerberg, Siddhi Jalmi, Jana Hajslova, Vera Schulzova, Folke Sitbon*

[#] These authors have contributed equally to this work

* Corresponding Author: folke.sitbon@slu.se

SUPPLEMENTARY FIGURES 1-16

FIGURE S1. Outline of sterol and SGA biosynthesis in potato.

FIGURE S2. OPLS-DA of metabolomic responses in potato tubers after wounding.

FIGURE S3. OPLS-DA of metabolomic responses potato tubers after light exposure.

FIGURE S4. General appearance of potato tubers after light exposure.

FIGURE S5. Calystegine levels in potato tubers after wounding or light exposure.

FIGURE S6. PCA of gene expression patterns in tubers after wounding or light exposure.

FIGURE S7. Differential gene expression in tubers after wounding or light exposure.

FIGURE S8. Gene expression profiling of tubers after wounding or light exposure.

FIGURE S9. Gene ontology of gene expression in tubers after wounding or light exposure.

FIGURE S10. Cluster analysis of gene expression in tubers after wounding or light exposure.

FIGURE S11. QPCR analysis of *GAME9* expression in Bintje and Magnum Bonum.

FIGURE S12. GAME9 cDNA sequence and amino acid composition in Bintje and Magnum Bonum.

FIGURE S13. QPCR validation of transcriptomic results.

FIGURE S14. Relative gene expression of *StHMGR*1 and *StTAM2* in transgenic Bintje plants.

FIGURE S15. Leaf SGA levels in *StTAM2*-overexpressing Desiree plants.

FIGURE S16. Model for light-regulated SGA pathway differences in Magnum Bonum vs Bintje tubers.

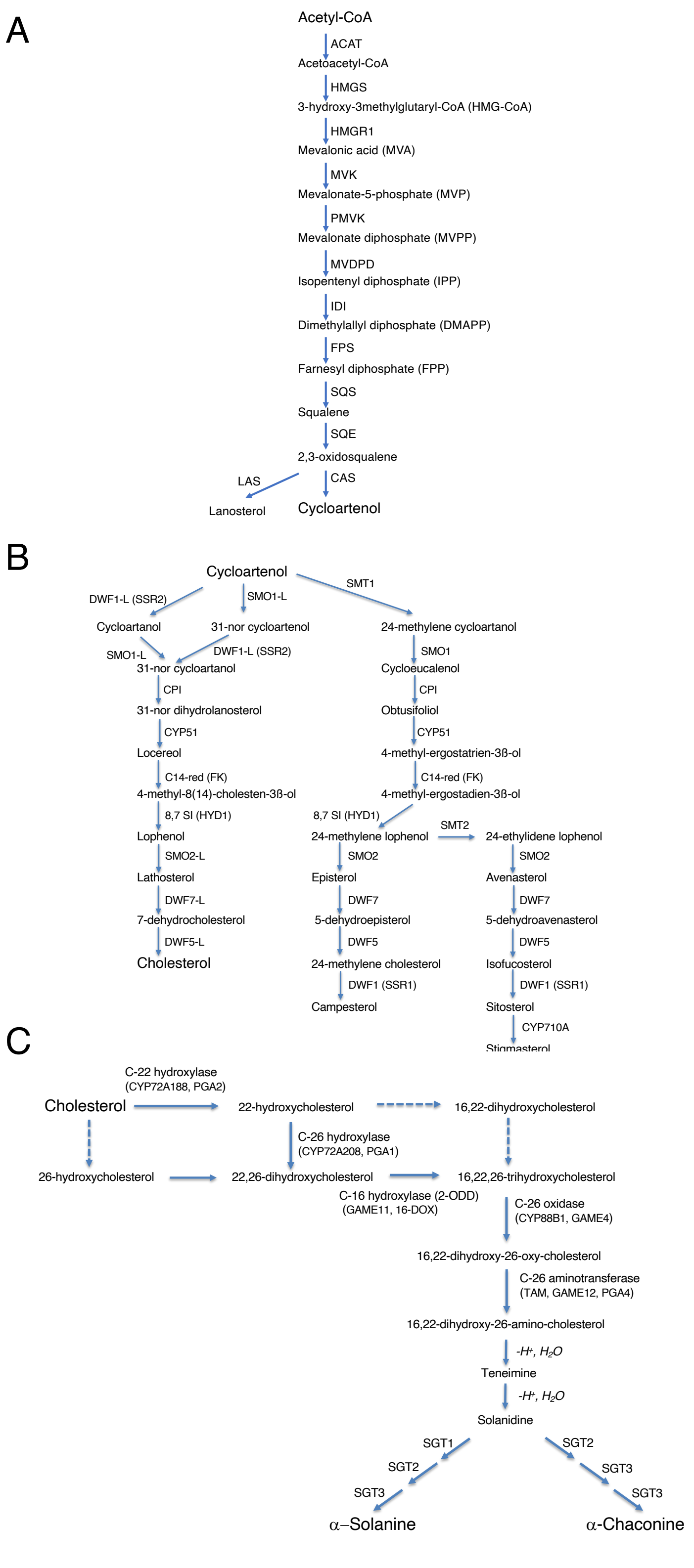

FIGURE S1. Outline of sterol and SGA biosynthesis in potato plants. **(A)** Cycloartenol synthesis from acetyl-CoA. **(B)** Cholesterol synthesis from cycloartenol. **(C)** SGA synthesis from cholesterol. Enzyme and gene abbreviations are listed in Table S1.

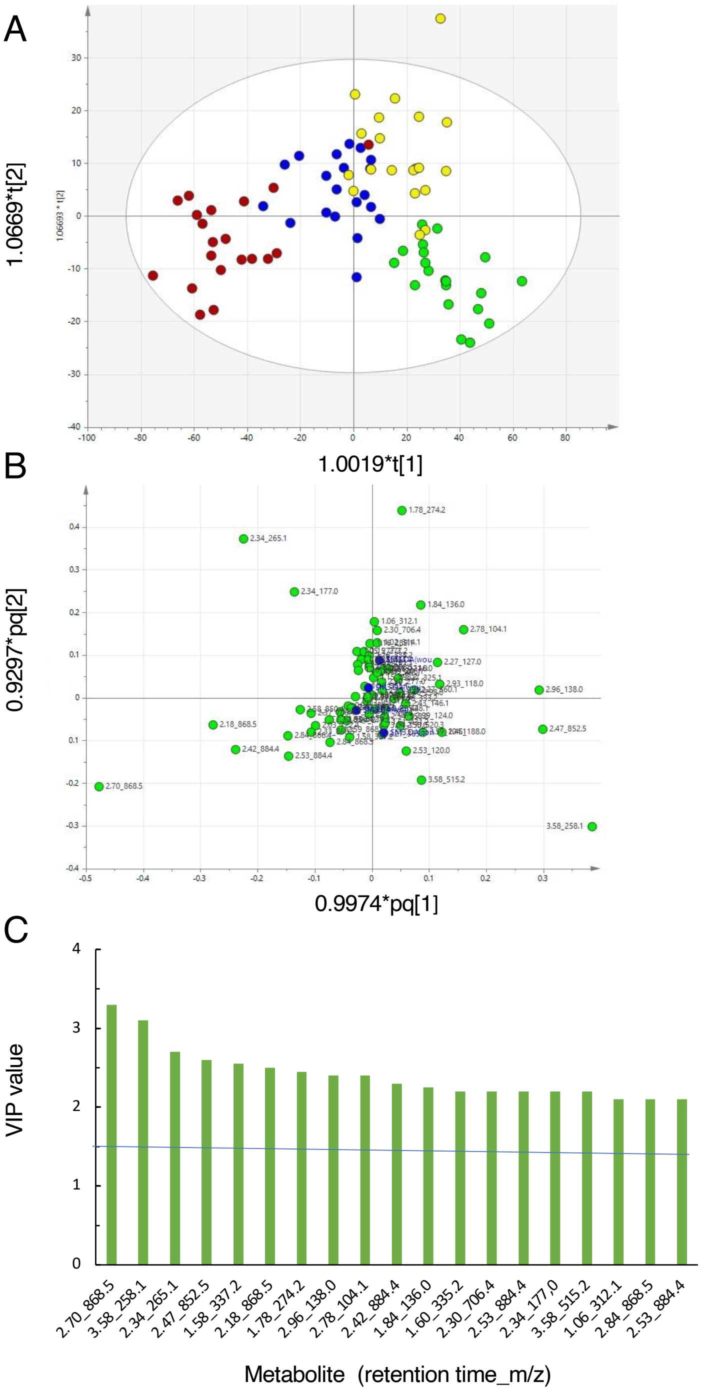

FIGURE S2. General metabolic responses in tubers from six potato cultivars subjected to wounding. Tubers were obtained from one or two years of cultivation, stored or not during 6 months, and treated or not by wounding. **(A)** Orthogonal partial least-square discriminant analysis (OPLS-DA) score scatter plot of the first two predictive components of metabolite analyses after wounding for 0 h (green), 12 h (yellow), 24 h (blue), or 48 h (red). **(B)** Corresponding loading scatter plot for peak areas in LC-MS (green circles). A set of quality control samples were run in parallel (blue circles). **(C)** Plot of features with a variable importance in projection (VIP) value > 1,5. Model statistics: R2X=0.64; R2Y=0.60; Q2=0.41; ellipse indicates Hotelling´s T2 (95 %).

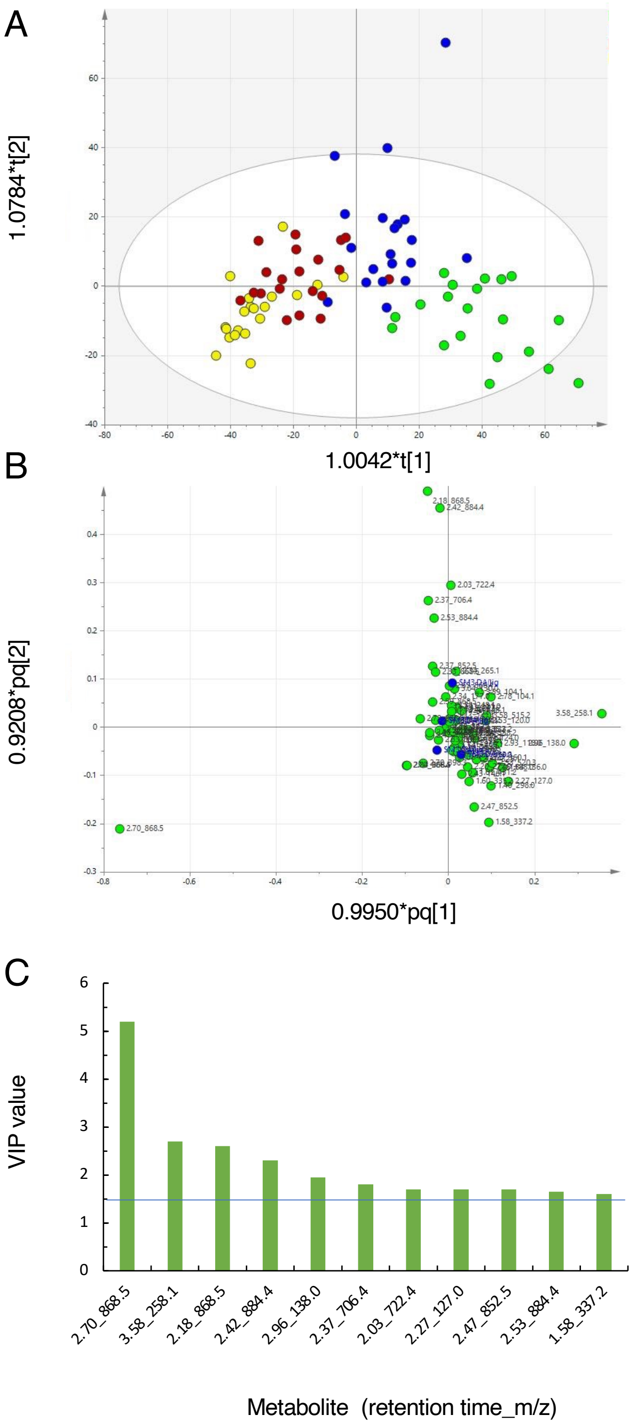

FIGURE S3. General metabolic responses in tubers from six potato cultivars subjected to light exposure. Tubers were obtained from one or two years of cultivation, stored or not for 6 months, and treated or not by light exposure (white fluorescent light; 100 µmol m^{-2} s^{-1}). **(A)** OPLS-DA score scatter plot of the first two predictive components of metabolite analyses after light exposure for 0 d (green), 2 d (blue), 4 d (red), or 8 d (yellow). **(B)** Corresponding loading scatter plot for peak areas in LC-MS (green circles). A set of quality control samples were run in parallel (blue circles). **(C)** Plot of features with a variable importance in projection (VIP) value >1.5. Model statistics: R2X = 0.56; R2Y =0.42; Q2=0.27; ellipse indicates Hotelling´s T2 (95 %).

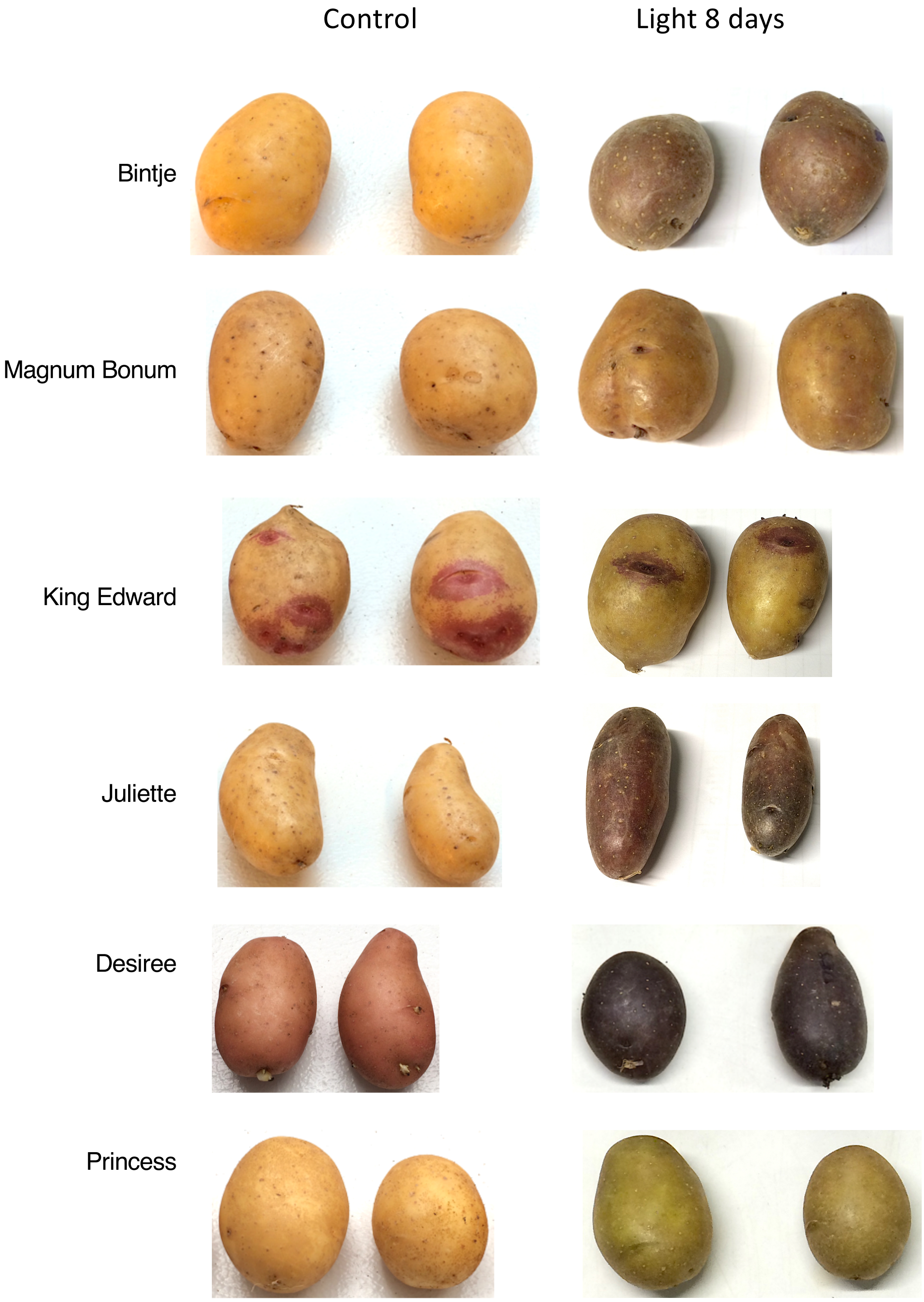

FIGURE S4. General appearance of tubers from six potato cultivars subjected to light exposure. Potato tubers were after harvest kept in darkness for two weeks (control), and then exposed to constant white fluorescent light (100 µmol m^{-2} s^{-1}) for 8 days in a controlled light cabinet. The red colour at harvest of the Desiree skin, and the red zones of King Edward, are normal for the cultivars. Pictures do not reflect size differences between cultivars.

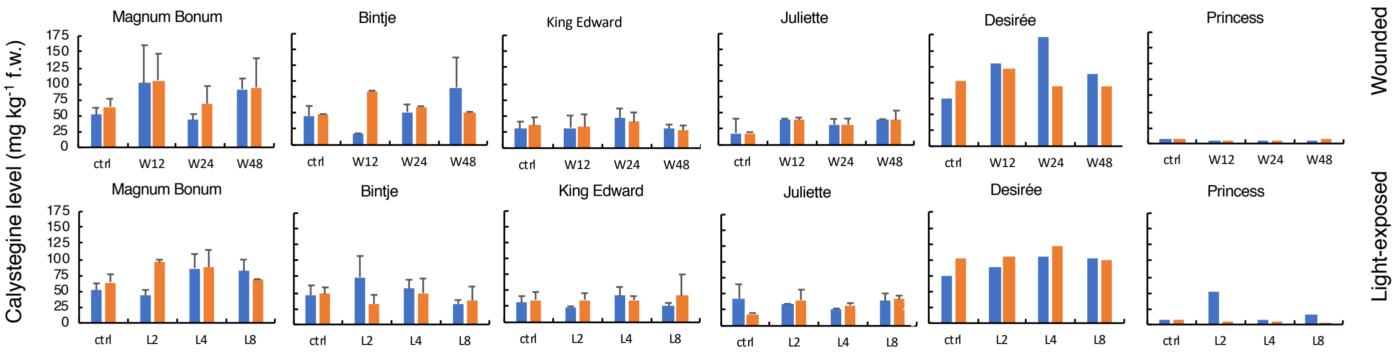

FIGURE S5. Calystegine alkaloid levels in tubers from six potato cultivars subjected to wounding or light exposure. Total calystegine levels were in a two-year study analyzed by LC-MS in tubers at time zero, or at different time points after wounding (12 h, 24 h, and 48 h; upper row) or white light exposure (2 d, 4 d, and 8 d; lower row), either at harvest (blue bars) or after a cold storage for 6 months in darkness (yellow bars). Total levels were calculated from the sum of calystegines A_3, B_2, and B_4. Mean level of total calystegine ± range of two years (MB, Bi, Ju, KE), or one year (De, Pr).

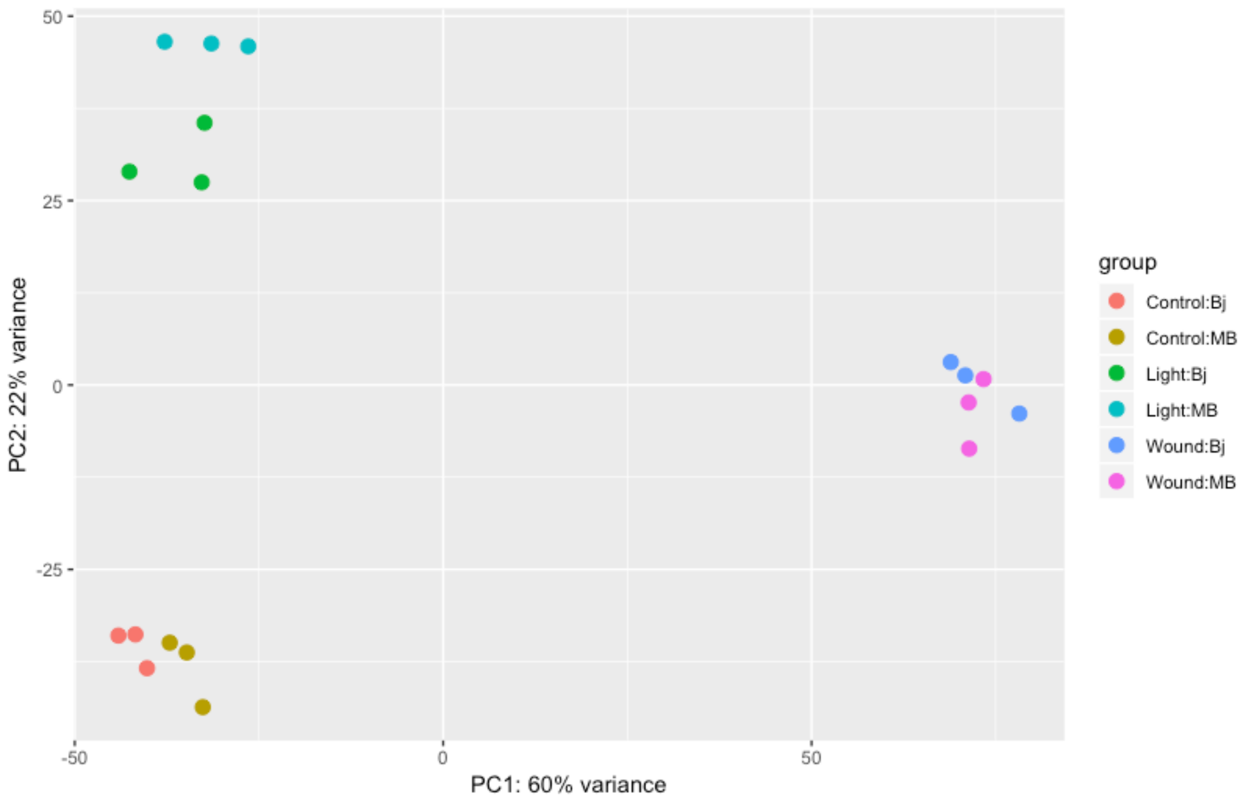

FIGURE S6. Principal component analysis (PCA) of gene expression patterns in tubers from the potato cultivars Bintje and Magnum Bonum after wounding or light exposure. Gene expression was determined by RNA sequencing of tubers from potato cultivars Bintje (Bj) and Magnum Bonum (MB), that had been wounded or exposed to white light for 24 h and 48 h, respectively. Control treatments indicate un-treated tubers analyzed at harvest. Colours indicate different treatments. Percentages of variation explained by each principal component (PC) are indicated along the axes.

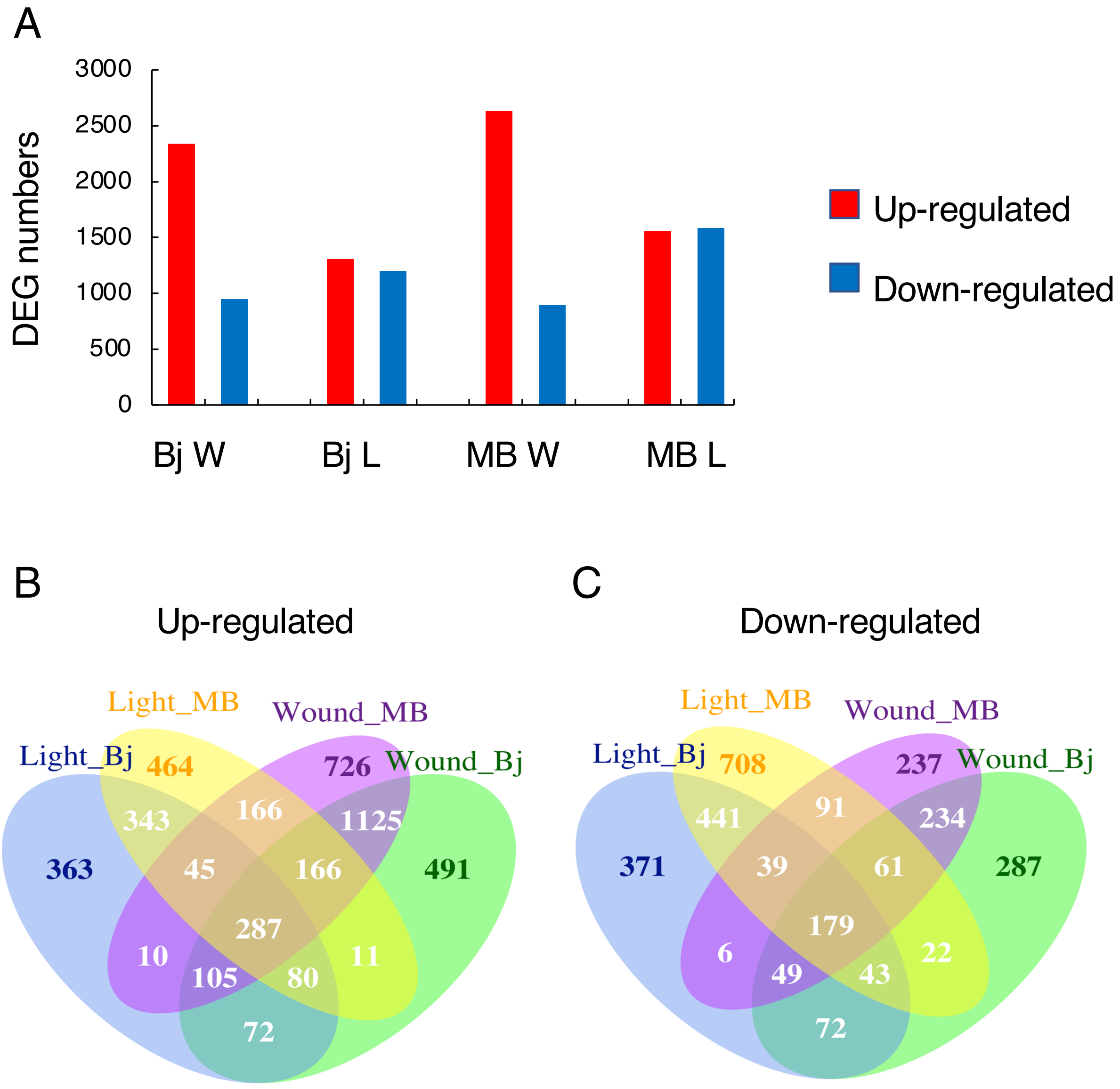

FIGURE S7. Differential gene expression in tubers from the potato cultivars Bintje and Magnum Bonum after wounding or light exposure. **(A)** Numbers of differentially expressed genes as determined by RNA sequencing of tubers from the potato cultivars Bintje (Bj) and Magnum Bonum (MB), that had been wounded or exposed to white light for 24 h and 48 h, respectively. **(B)** Venn display of up-regulated genes in the two cultivars. **(C)** Venn display of down-regulated genes in the two cultivars.

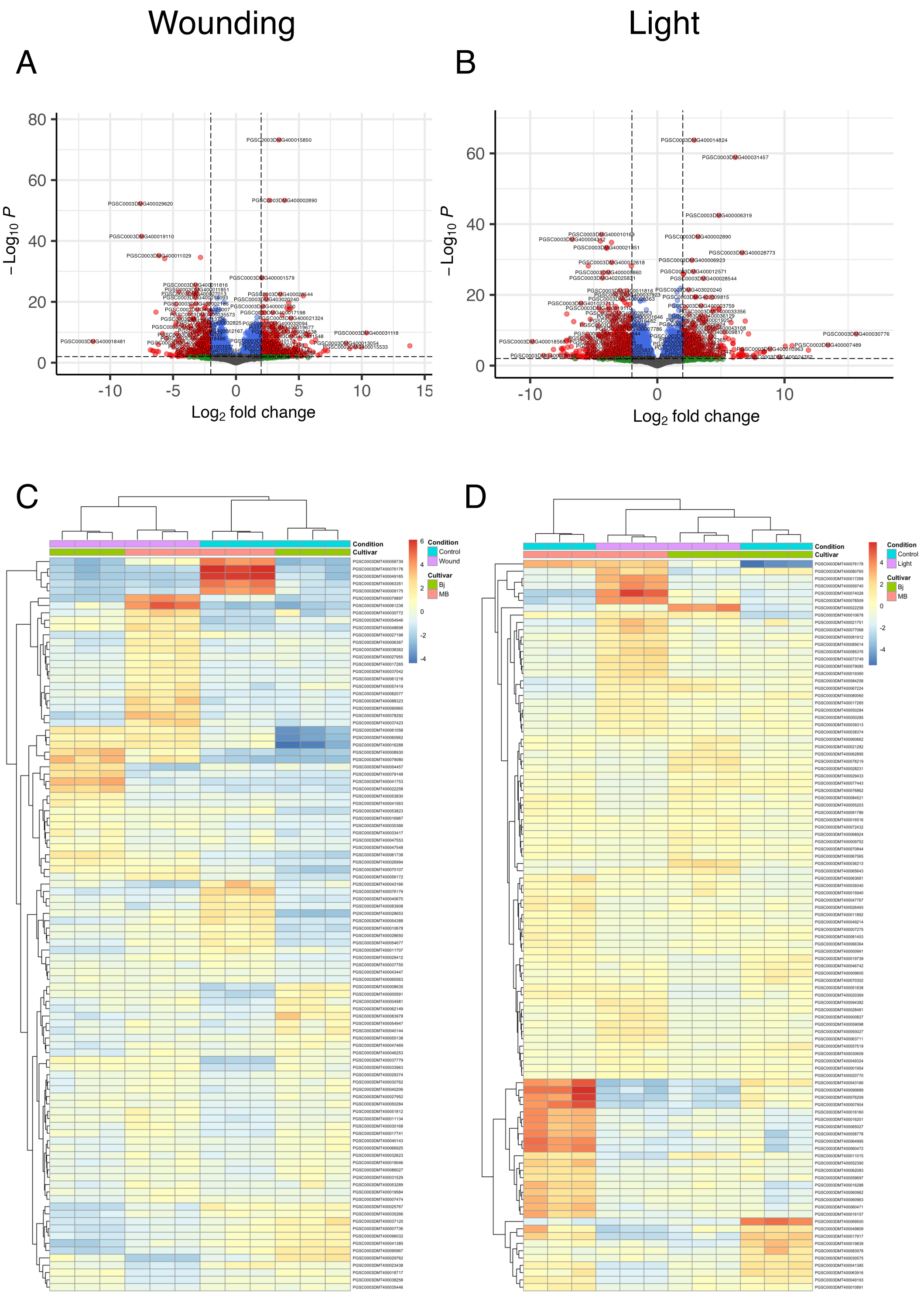

FIGURE S8. Gene expression profiling in tubers from the potato cultivars Bintje and Magnum Bonum after wounding or light exposure. Tubers were treated by wounding (24 h), or light exposure (48 h). Volcano plots of statistical significance against $\log_2$ of expression fold-change after wounding **(A)** or light exposure **(B)**. HeatMap displays of 100 transcripts having the most significant P-values for the gene x cultivar interaction after wounding **(C)** or light exposure **(D)**. Full lists of genes are given in Tables S7 and S8.

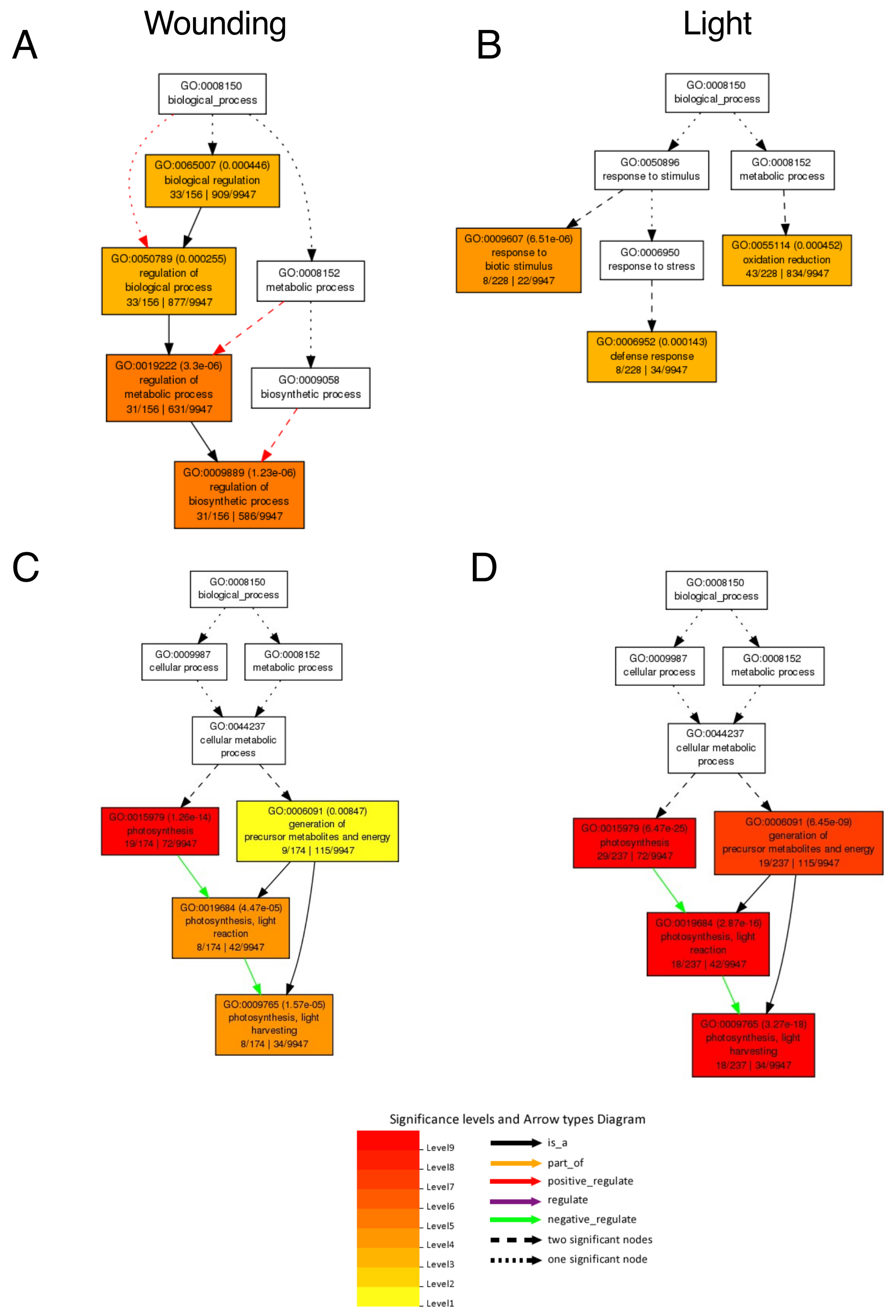

FIGURE S9. Gene onthology (GO) enrichment analysis of differentially expressed genes in tubers from the potato cultivars Bintje and Magnum Bonum after wounding or light exposure. Genes with significant response differences in an RNAseq analysis of tubers after a wounding or light treatment (Padj<0.01, and a fold-change >4), were used as input to identify the most enriched biological processes comparing the two cultivars, using the AgriGO Toolkit database v2.0 (FDR < 0.05). The full list of transcripts (RPKM> 20) detected in Bintje control tubers were used as background reference in all analyses. GO enrichment analysis in the biological process category of genes with higher expression in Magnum Bonum than Bintje for wounded **(A)**, or light-exposed **(B)**, tubers. GO enrichment analysis in the biological process category of genes with higher expression in Bintje than Magnum Bonum, in wounded **(C)**, or light-exposed **(D)**, tubers. Only the most enriched processes are shown, and the most significant results are indicated in red. GO term number, p-value, and GO term are indicated.

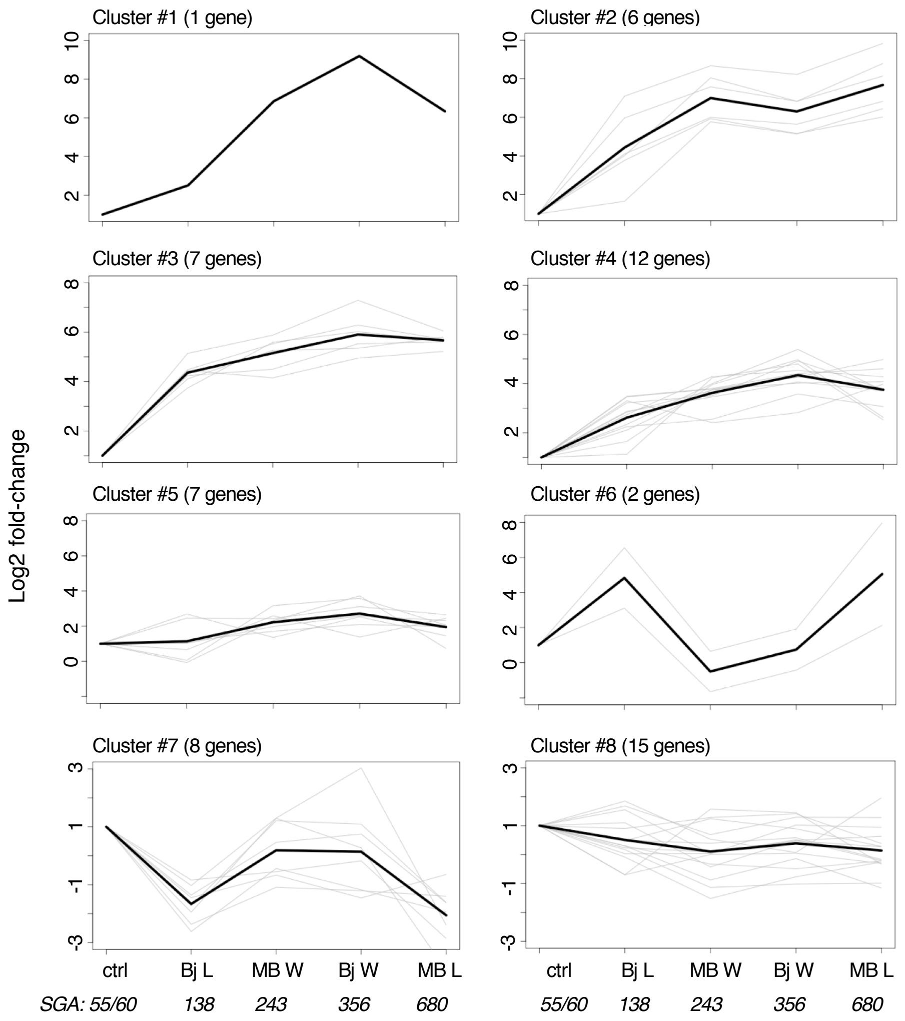

FIGURE S10. Clustered gene expression patterns of 58 sterol- and SGA-related genes in tubers from the potato cultivars Bintje and Magnum Bonum after wounding or light exposure. The same set of 58 genes as in Fig. 3 are displayed. Average RPKM fold-change values relative to untreated control samples were subjected to k-means clustering into 8 different clusters based on the relation to the end-point SGA levels in the experiment. Gray lines represent individual induction values of each transcript in a cluster, black lines show the median induction pattern for each cluster. Ctrl, control; Bj, Bintje; MB, Magnum Bonum. SGA indicate the total SGA levels (mg kg^{-1} FW) measured in untreated control tubers (Bj/MB) or in tubers 48h after wounding (W) or 8 days after light exposure (L) as described in Fig. 2. The genes in each cluster are listed in Table S9.

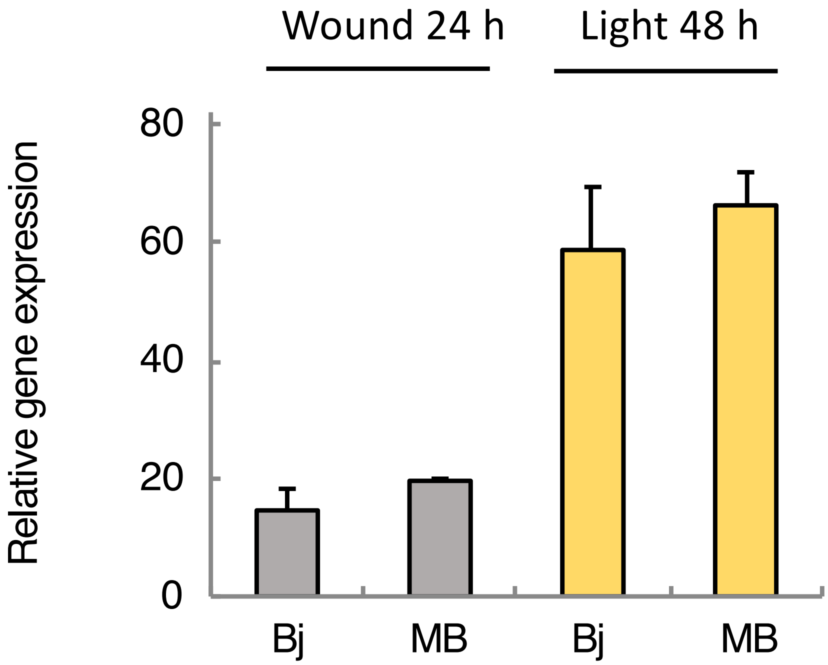

FIGURE S11. Relative gene expression levels of the transcription factor *StGAME9* in tubers from the potato cultivars Bintje and Magnum Bonum after wounding or light exposure. Pools of four tubers from cultivars Bintje (Bj) and Magnum Bonum (MB), were at harvest during two cultivation years subjected to a wounding (24 h) or light exposure (48 h) treatment. Mean transcript levels ± range of the two years.

```
Bintje          MNIAIDDDEIFSLPSLDELESITHLLYDDDSDFFETLSPMSLDSTTLLPNNPTPNSLESP
Magnum_Bonum    MNIAIDDDEIFSLPSLDELESITHLLYDDDSDFFETLSPMSLDSTTLLPNNPTPNSLESP
Solyntus        MNISIDDDEIFSLPSLDELESITHLLYDDDSDFFETLSPMSLDSTTLLPNNPTSNSLESP
Phureja         MNIAIDDDEIFSLPSLDELESITHLLYDDDSDFFETLSPMSLDSTTLLPNNPTPNSLESP
Otava           MNIAIDDDEIFSLPSLDELESITHLLYDDDSDFFETLSPMSLDSTTLLPNNPTPNSLESP
                ***:*************************************************.******

Bintje          VRPEGTKETFVAREHEESAPQDWRRFIGVRRRQWGTFSAEIRDPNRRGARLWLGTYESPQ
Magnum_Bonum    VRPEGTKETFVAREHEESAPQDWRRFIGVRRRQWGTFSAEIRDPNRRGARLWLGTYESPQ
Solyntus        VRPEGTKETSVARERECSAPQDWRRFIGVRRRQWGTFSAEIRDPNRRGARLWLGTYESPQ
Phureja         VRPEGTKETFVAREHEESAPQDWRRFIGVRRRQWGTFSAEIRDPNRRGARLWLGTYESPQ
Otava           VRPEGTKETFVAREHEESAPQDWRRFIGVRRRQWGTFSAEIRDPNRRGARLWLGTYESPQ
                ********* ****:*********************************************
```

AP2/ERF domain

```
Bintje          DAALAYDQAAYKIRGTKARLNFPDLIGSDVPMPPRVTARRRTRSRSRSPEPSTTSSSSSS
Magnum_Bonum    DAALAYDQAAYKIRGTKARLNFPDLIGSDVPMPPRVTARRRTRSRSRSPEPSTTSSSSSS
Solyntus        DAALAYDQAAYKIRGTKARLNFPDLIGSDVPMPPRVTARRRTRSRSRSPEPSTTSSSSSS
Phureja         DAALAYDQAAYKIRGTKARLNFPDLIGSDVPMPPRVTARRRTRSRSRSPEPSTTSSSSSS
Otava           DAALAYDQAAYKIRGTKARLNFPDLIGSDVPMPPRVTARRRTRSRSRSPEPSTTSSSSSS
                ************************************************************
```

AP2/ERF domain

```
Bintje          SSSSSSSSSLENGTKKRKIDLINSIAKAKLLCGVNLQMLIQM------
Magnum_Bonum    SSSSSSSSSLENGTKKRKIDLINSIAKAKLLCGVNLQMLIQM------
Solyntus        SSSSSSSSSLENGTKKRKIDLINSIAKAKLLCGVNLQMLIQM------
Phureja         SSSSSSSSSMENGTKKRKIDLINSIAKAKLLCGVNLQMLIQM------
Otava           SSSSSSSSSLENGTKKRKIDLINSIAKAKLLCGGGATSVLEVDPNFSL
                *********:*********************** .   ::::
```

FIGURE S12. Amino acid sequence alignment of GAME9 from different potato genotypes. The full coding sequence for GAME9 was amplified by RT-PCR from RNA extracted from Bintje and Magnum Bonum tubers and sequenced. Corresponding sequences from cvs. Solyntus (CP055241), Otava (KAH0670900), and the wild potato species *S. Phureja* (M1CGB0), were retrieved from NCBI and included in the alignment. Residues conserved in all five proteins are indicated with an asterisk (*), conservative and semi-conservative amino acid changes are indicated with a colon (:), or a dot (.), respectively. The conserved GAME9^{135A} amino acid is highlighted in yellow. The AP2/ERF DNA-binding domain characteristic of the ERF family is underlined.

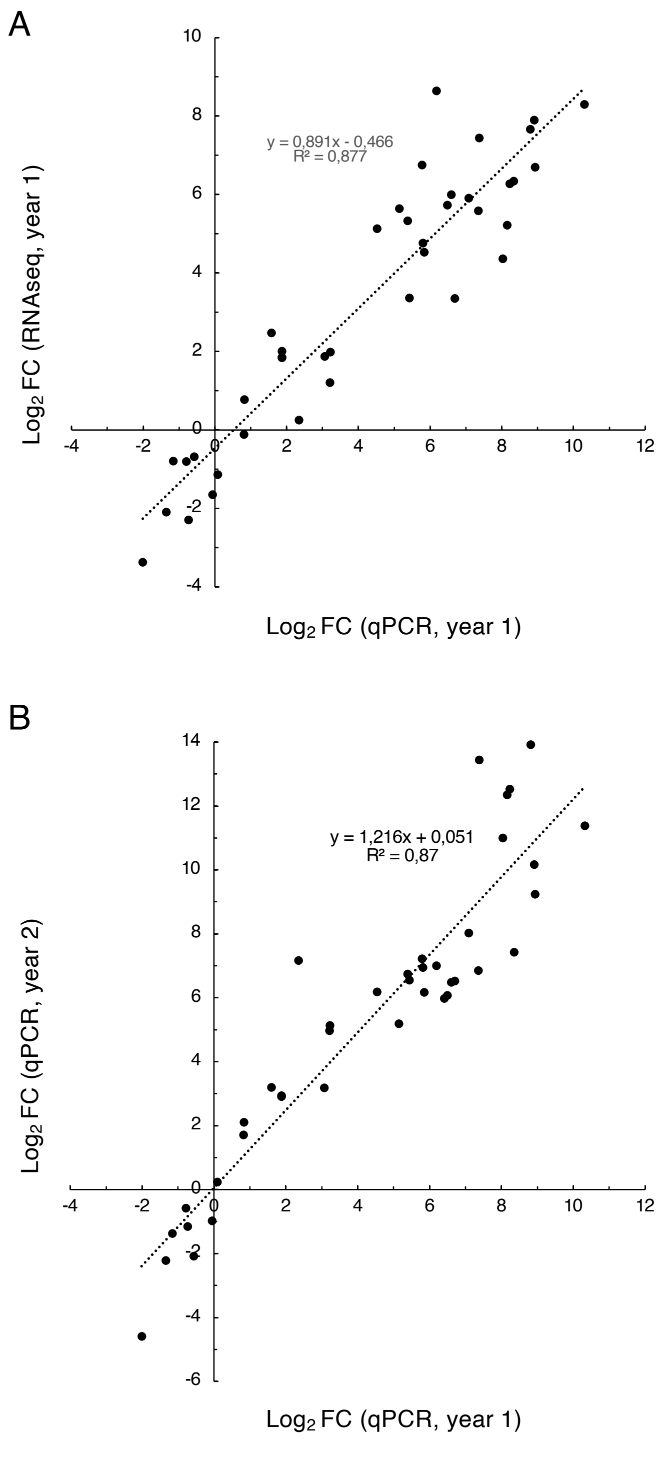

FIGURE S13. Validation of gene expression analyses. **(A)** Gene expression of ten selected genes with a role in sterol and/or SGA metabolism were analyzed by QPCR in tubers from potato cultivars Bintje and Magnum Bonum, that had been subjected to a 24-h wounding, or to a 48-h light exposure treatment. The $\log_2$ of gene expression relative to un-treated controls (log fold-change; FC) was compared to the corresponding values obtained by RNAseq analysis from the same samples (average value from triplicate analyses). **(B)** The $\log_2$ of gene expression FC in wounded and light-exposed tubers were compared to similarly treated tubers from an experimental repetition the following year.

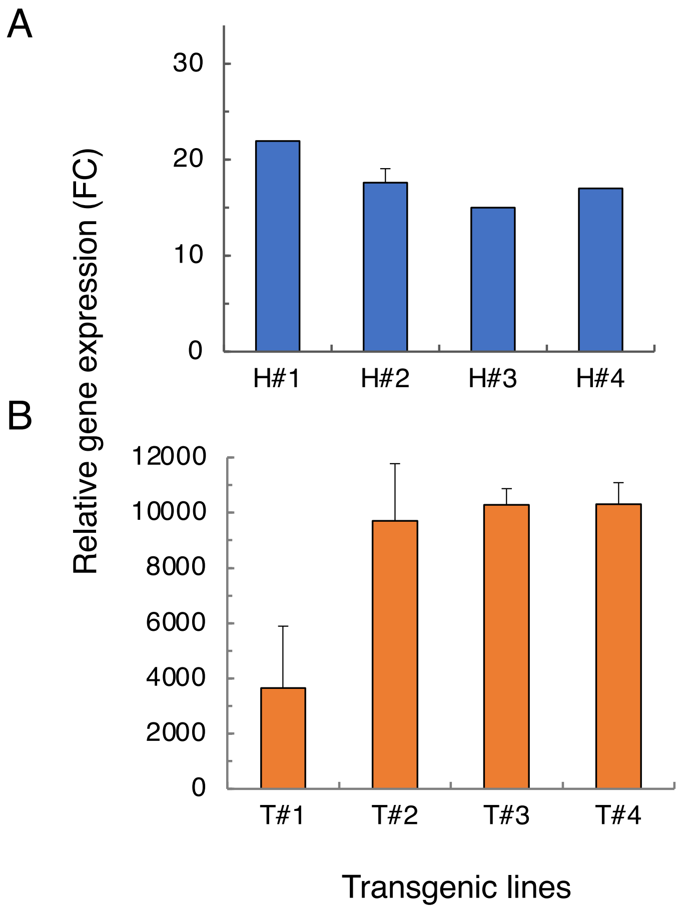

FIGURE S14. Relative transgene expression levels in leaf samples from wild-type potato plants (cv Bintje) and derived 35S*:StHMGR1$_{MB}$* and 35S:*StTAM2$_{MB}$* transformants. **(A)** Transgene expression in leaves of 35S:*StHMGR1$_{MB}$* transgenic lines H1 to H4. **(B)** Transgene expression in leaves of 35S:*StTAM2$_{MB}$* transgenic lines T1 to T4. Gene expression was monitored by QPCR analysis, and Ct values of the target gene were normalized against the *TUBULIN* reference gene. The transcript expression level relative to its basal expression in the Bintje wild type was calculated according to Livak & Schmittgen (2001). Mean ± range or SD of triplicate QPCR analyses of the plants used for the corresponding SGA analyses in Figure 6.

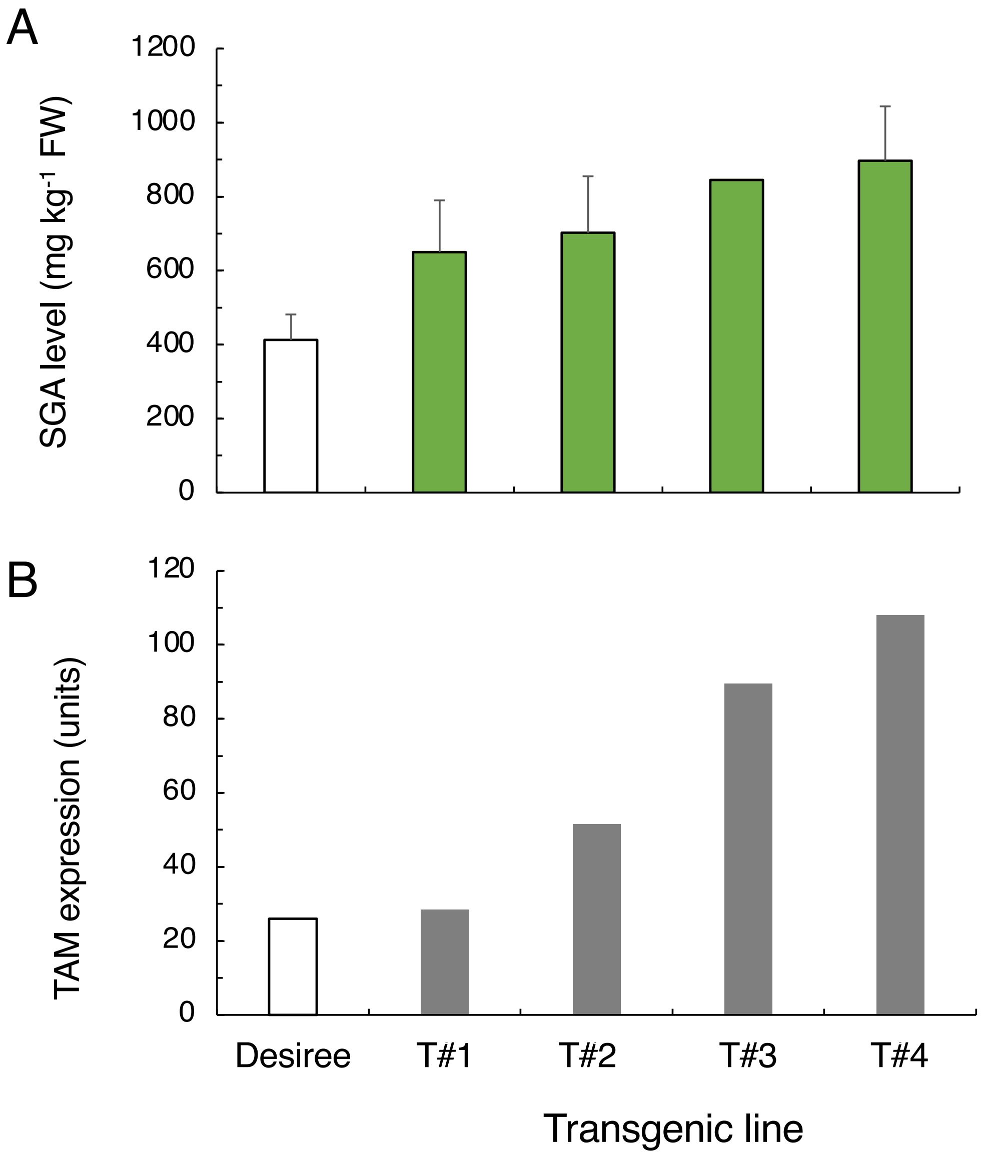

FIGURE S15. Leaf SGA levels and transgene expression in wild-type potato Desiree and derived 35S:*StTAM2*$_{Ken}$ transformants. A, Mean SGA level ± SD or range, from the sum of α-chaconine and α-solanine as measured by LC/MS, in wild-type Desiree plants (n=5 plants; white bars), and 35S:*StTAM2*$_{Ken}$ transformants overexpressing a *StTAM2* cDNA from cv. Kennebec (n=1, or 2 plants per line; green bars). Lines with the four highest SGA values among 8 independent lines analyzed are shown. **(A)** statistical difference between thes transformants and the wild type was significant at $p<0.001$ (Student´s *t*-test). **(B)** *StTAM2*$_{Ken}$ expression as analyzed by RT-PCR from equal amounts of leaf total RNA, and a digital scan of amplified products on an agarose gel.

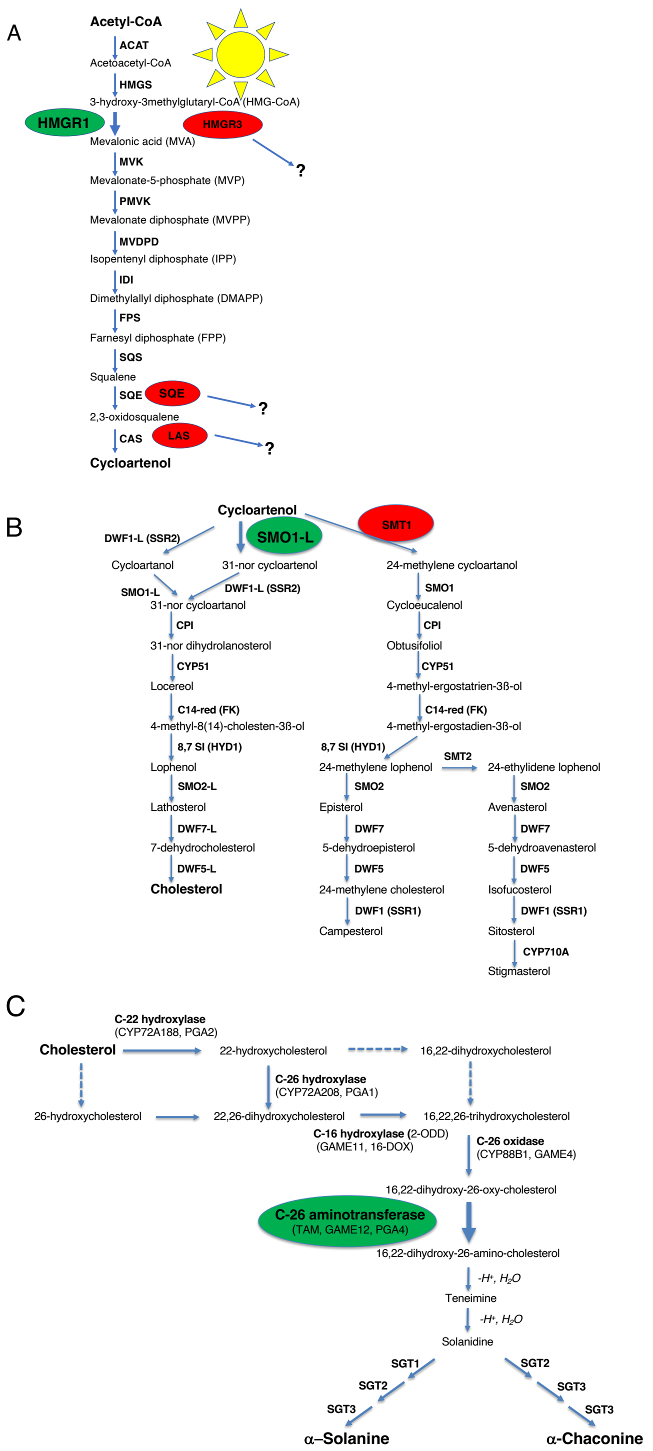

FIGURE S16. Model for SGA-biosynthetic differences between Magnum Bonum and Bintje tubers during light exposure. Light exposure leads to significantly higher SGA levels in Magnum Bonum than in Bintje, at least in part due to a stronger induction of genes that encode limiting enzymes (green circles) in the cycloartenol **(A)**, cholesterol **(B)**, and SGA **(C)** biosynthesis pathways, together with a stronger down-regulation of genes that encode enzymes in competing steroidal reactions (red circles). Question marks indicate uncharacterized competing products.

REFERENCES

Livak, K.J., Schmittgen, T.D. (2001). Analysis of relative gene expression data using real-time quantitative PCR and the $2^{-\Delta\Delta CT}$ method. *Methods* 25, 402-408.
